# A Neuroimaging Signature of Cognitive Aging from Whole‐Brain Functional Connectivity

**DOI:** 10.1002/advs.202201621

**Published:** 2022-07-10

**Authors:** Rongtao Jiang, Dustin Scheinost, Nianming Zuo, Jing Wu, Shile Qi, Qinghao Liang, Dongmei Zhi, Na Luo, Young‐Chul Chung, Sha Liu, Yong Xu, Jing Sui, Vince Calhoun

**Affiliations:** ^1^ Department of Radiology and Biomedical Imaging Yale School of Medicine New Haven CT 06520 USA; ^2^ Interdepartmental Neuroscience Program Yale University New Haven CT 06520 USA; ^3^ Department of Statistics and Data Science Yale University New Haven CT 06520 USA; ^4^ Child Study Center Yale School of Medicine New Haven CT 06510 USA; ^5^ Brainnetome Center and National Laboratory of Pattern Recognition Institute of Automation Chinese Academy of Sciences Beijing 100190 P. R. China; ^6^ School of Artificial Intelligence University of Chinese Academy of Sciences Beijing 100049 P. R. China; ^7^ Department of Medical Oncology Beijing You‐An Hospital Capital Medical University Beijing 100069 P. R. China; ^8^ College of Computer Science and Technology Nanjing University of Aeronautics and Astronautics Nanjing 211106 P. R. China; ^9^ Department of Biomedical Engineering Yale University New Haven CT 06520 USA; ^10^ State Key Laboratory of Cognitive Neuroscience and Learning Beijing Normal University Beijing 100088 P. R. China; ^11^ Department of Psychiatry Jeonbuk National University Medical School Jeonju 54907 Republic of Korea; ^12^ Department of Psychiatry Chonbuk National University Hospital Jeonju 54907 Republic of Korea; ^13^ Department of Psychiatry and MDT Center for Cognitive Impairment and Sleep Disorders First Hospital First Clinical Medical College of Shanxi Medical University Taiyuan 030001 P. R. China; ^14^ Tri‐institutional Center for Translational Research in Neuroimaging and Data Science (TReNDS) Georgia Institute of Technology Emory University and Georgia State University Atlanta GA 30303 USA

**Keywords:** biomarker, brain age, cognition aging, individualized prediction, predictive neuroimaging

## Abstract

Cognitive decline is amongst one of the most commonly reported complaints during normal aging. Despite evidence that age and cognition are linked with similar neural correlates, no previous studies have directly ascertained how these two constructs overlap in the brain in terms of neuroimaging‐based prediction. Based on a long lifespan healthy cohort (CamCAN, aged 19–89 years, *n* = 567), it is shown that both cognitive function (domains spanning executive function, emotion processing, motor function, and memory) and human age can be reliably predicted from unique patterns of functional connectivity, with models generalizable in two external datasets (*n* = 533 and *n* = 453). Results show that cognitive decline and normal aging both manifest decrease within‐network connections (especially default mode and ventral attention networks) and increase between‐network connections (somatomotor network). Whereas dorsal attention network is an exception, which is highly predictive on cognitive ability but is weakly correlated with aging. Further, the positively weighted connections in predicting fluid intelligence significantly mediate its association with age. Together, these findings offer insights into why normal aging is often associated with cognitive decline in terms of brain network organization, indicating a process of neural dedifferentiation and compensational theory.

## Graphical abstract

Here it is reported that age and multiple cognitive functions can be predicted from whole‐brain functional connectome. Moreover, cognitive decline and normal aging both manifest decreased within‐network connections (especially default mode and ventral attention networks) and increased between‐network connections (somatomotor network), indicating a process of neural dedifferentiation and compensational theory.

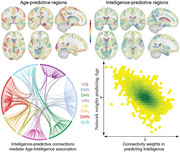

## 1 Introduction

Currently available evidence from cognitive neuroscience has consistently found that normal aging is accompanied by a progressive decline in cognition, especially in domains of processing speed, working memory, and executive function.^[^ ^1^, ^2^, ^3^ ^]^ Nonetheless, the rate of age‐associated cognitive changes is not inevitably uniform, but instead, pronounced heterogeneity exists.^[^ ^4^, ^5^ ^]^ Understanding the basis of cognition decrement over the course of healthy aging is of crucial relevance for delivering effective interventions to combat and even reverse age‐accompanied cognitive decline.

Meanwhile, healthy aging is revealed to be associated with extensive changes to the structure and function of the human brain even in the absence of overt disease, which are characterized by an increase in grey matter atrophy, cortical thinning, white matter volume loss, and alterations in structural and functional connectivity.^[^ ^6^, ^7^, ^8^ ^]^ There is ample evidence that individual brain regions follow temporally distinct trajectories across the full lifespan, with age associations appearing to be stronger for some regions than others.^[^ ^9^, ^10^, ^11^ ^]^ In this sense, the regional heterogeneity in aging trajectories, when considered in isolation, poses immense challenges in accurately characterizing patterns of brain aging.^[^ ^12^, ^13^ ^]^ Machine learning is emerging as a promising tool to distill a rich set of distributed imaging features into a single index whereby accurate prediction of biological age can be achieved from unseen individuals. Potentially most exciting is the demonstration that the brain‐predicted age is linked with multiple physical and cognitive aspects, and sensitive to subtle brain changes occurring before outward manifestations of neurodegenerative diseases, providing an innovative biomarker of brain health.^[^ ^14^, ^15^ ^]^

Despite the potential, some key issues remain unaddressed. First, relative to the abundance of studies using structural MRI features, attempts employing functional measurements to predict brain age have been few.^[^ ^16^, ^17^ ^]^ This knowledge gap is surprising given that 1) growing evidence implies that changes in the brain's functional organization precede changes in anatomy;^[^ ^18^ ^]^ 2) functional measurements have higher relevance to cognition and are more likely disrupted in developmental disorders.^[^ ^19^ ^]^ Second, most studies concern more on improving prediction accuracy or testing the feasibility of brain age model in a cascade of psychiatric disorders than interpreting the predictive neuroimaging signatures, which can hamper gaining clinically and biologically meaningful insights into the underlying mechanisms involved.^[^ ^20^ ^]^ Third, the utility of brain‐predicted age is generally established by probing its relevance to cognitive variables and revealing that having an older‐appearing brain is associated with poorer cognitive fitness.^[^ ^21^, ^22^ ^]^ It is underexplored regarding how cognitive development and age overlap in the brain by assessing their common and distinct neural representations in the context of predictive modeling. This knowledge gap hinders efforts to locate brain targets for early interventions that could aid in developing personalized neuroprotective treatments.

**Figure 1:**
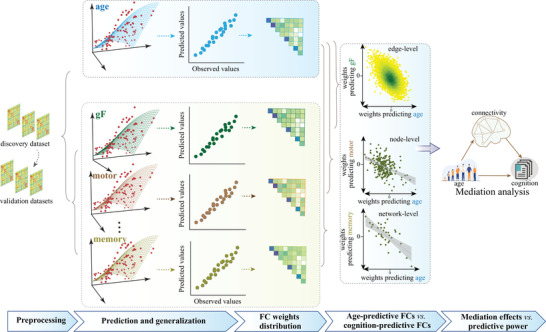
Flowchart of the main analyses. To examine whether normal aging and cognitive function have common neural representations, we developed separate predictive models for brain age and eight cognitive metrics based on whole‐brain connectome features in a cross‐sectional adult lifespan sample (aged 19–89 years), and compared their weight maps at connection, node, and network level.

In the present study, we exploited a connectome‐based machine learning approach within fully cross‐validation (CV) analyses to probe reliable and robust imaging signatures for eight distinct cognitive functions that significantly decline over aging and brain age from whole‐brain functional connectivity (**Figure** **1**). The validity of identified biomarkers was corroborated by testing their generalizability in two other external, heterogenous datasets. Crucially, extraction and comparison of the predictive brain signatures and their corresponding weight maps allow us to quantify the extent to which cognitive development and normal aging overlap in the brain.

## 2 Results

### 2.1 Connectome‐Based Prediction of Brain Age and Multiple Cognitive Scores

**Figure 2:**
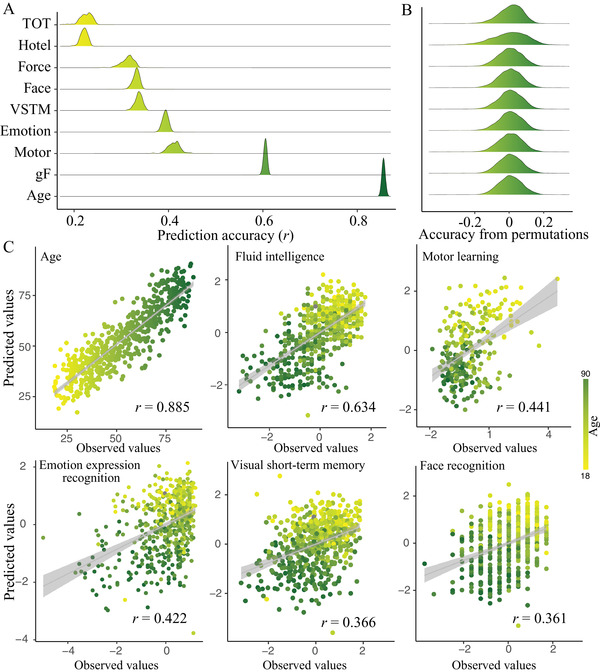
Connectome‐based prediction results for age and eight cognitive metrics spanning domains of executive function, emotional processing, motor function, and memory. A) Distribution of prediction accuracies across 200 repetitions of cross‐validation. B) Distribution of accuracies based on permutation testing across 5000 iterations. C) Scatter plot shows prediction of age and five representative cognitive metrics. Although the prediction framework was repeated 200 times, we just show results from one iteration for visualization. *g*F, fluid intelligence; TOT, tip‐of‐tongue; VSTM, visual short‐term memory.

Based on whole‐brain connectivity features, we built a separate predictive model for age and eight cognitive measures within a repeated CV framework. **Figure** **2**A shows the distribution of prediction accuracies across 200 repetitions of CV for brain age and each of the eight cognitive metrics (*R* ^2^ distribution, Figure S1, Supporting Information). A permutation test revealed that all correlations were statistically significant at *p* < 2.0 × 10^–4^ (Figure 2B). Specifically, the strongest result was generated for age, with a mean correlation between actual versus predicted values reaching *r* = 0.885 ± 0.003 (*R* ^2^ = 0.78 ± 0.005, root mean squared error (RMSE) = 8.57 ± 0.10), averaging across all CV repetitions (Figure 2C). Among all cognitive metrics, fluid intelligence shows the highest predictability (*r* = 0.634 ± 0.003, *R* ^2^ = 0.40 ± 0.004, RMSE = 5.17 ± 0.02), with performance of the other metrics varying from *r*(TOT) = 0.255 ± 0.012 to *r*(motor learning) = 0.441 ± 0.013. Notably, prediction accuracies slightly decreased after controlling for mean framewise displacement (FD) but remained high enough to be significant (Table S1, Supporting Information). When constructing predictive models in a subset of subjects with low head motion (Figure S2, Supporting Information), we observed nearly comparable accuracies, suggesting that the predictive models are robust to head movements. Moreover, gender has little impact on the predictions.

### 2.2 Predictive Network Anatomy

**Figure 3:**
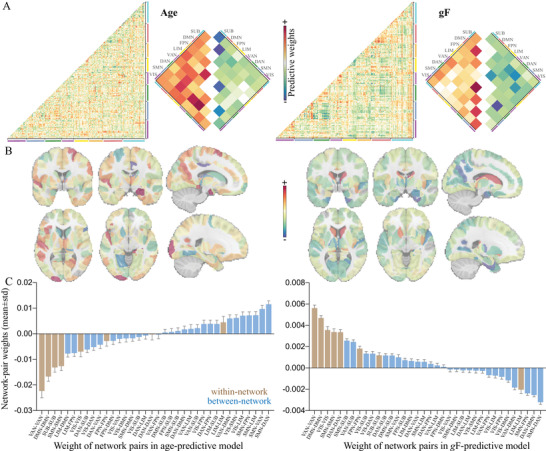
Distributions of weight maps in predicting age and fluid intelligence. A) Distributions of raw predictive weights averaged across 2000 cross‐validation rounds. The cell plots show the network‐level representation of the predictive weights. For each pair of networks (between‐network and within‐network), we averaged predictive weights of all connections belonging to that network pair. Positive weights and negative weights were separately summarized for each network pair to demonstrate their relative contribution. B) Distributions of weight maps at the node level. The node‐level representation was achieved by summarizing weight values of all connections incident to each of the 246 atlas‐defined functional macroscale regions. C) Mean weights distribution of within‐network and between‐network connections in age‐ and *g*F‐predictive models. Error bars indicate standard deviation. DAN, dorsal attention network; DMN, default mode network; FPN, frontoparietal network; *g*F, fluid intelligence; LIM, limbic network; SMN, somatomotor network; SUB, subcortical network; VAN, ventral attention network; VIS, visual network; VSTM, visual short‐term memory.

Since we leveraged the whole‐brain connectome to make predictions, each feature obtained a predictive weight representing its contribution. When summarizing the weight maps from low‐level connections (**Figure** **3**A) to high‐level networks, significant trends are revealed. Specifically, in the prediction of brain age, connections within default mode (DMN), ventral attention (VAN), somatomotor (SMN), and subcortical (SUB) demonstrated prominent negative weights; while connections within limbic (LIM) showed prominent positive weights. Moreover, positive predictive weights were also prominent in SMN‐connected between‐network connections with dorsal attention (DAN), VAN, and frontoparietal (FPN). As expected, these networks were also revealed to exhibit the greatest involvement in predicting cognitive functions, but in a completely opposite direction, that is, connections that grow in strength across aging would predict lower cognitive abilities (Figure 3B,C).

### 2.3 Overlap of Predictive Models between Brain Age and Cognitive Function during Normal Aging

**Figure 4:**
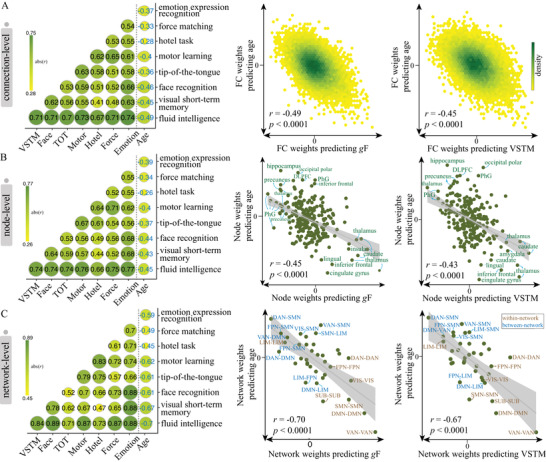
Overlap of predictive models between brain age and cognitive function during normal aging. To evaluate the extent to which predictive models were similar to or distinct from each other, we calculated the Pearson's correlation between the averaged weight maps from the age‐predictive model and each of the eight cognition‐predictive models at the A) connection (*n* = 30 135), B) node (*n* = 246), and C) network (*n* = 36) level. Scatter plots in each row are representations of the correlation between age‐predicted model and *g*F‐predictive/VSTM‐predictive model. Permutation test showed that all correlations were significant at *p* < 0.0001 across 10 000 iterations. DAN, dorsal attention network; DMN, default mode network; FPN, frontoparietal network; *g*F, fluid intelligence; LIM, limbic network; SMN, somatomotor network; SUB, subcortical network; VAN, ventral attention network; VIS, visual network; VSTM, visual short‐term memory.

At the connectivity level, models for age and cognitive function were inversely correlated, with the strongest effect observed between age and fluid intelligence (*r* = −0.49, permutation test *p* < 10^–5^, **Figure** **4**A). The negative correlations of connectome weights between age and cognitive measures aligned with their behavioral relationship. Node‐level analysis revealed virtually similar patterns with connection‐level results. Specifically, functional nodes gaining the highest positive power in predicting age and negative power in predicting fluid intelligence primarily included the right hippocampus, right precuneus, right dorsolateral prefrontal cortex, and right parahippocampus. Nodes making the most negative contributions to age prediction and positive contributions to intelligence prediction predominantly involved the right cingulate gyrus, right thalamus, left lingual gyrus, and the caudate (Figures 3B,4B; Table S2, Supporting Information).

When mapping whole‐brain connections into network‐level representations, we observed a general elevated correlation between age‐ and cognition‐predictive models, with the strongest effect again observed between age and fluid intelligence (*r*_age‐_ *_g_*_F_ = −0.70, permutation test *p* <10^–5^, Figure 4C). Specially, the age‐ and *g*F‐predictive models demonstrated the highest similarity of weight patterns in SUB, DMN, VAN, and SMN (Figure S3, Supporting Information). A further examination of these predictive networks revealed some intriguing trends. Since the cognition‐predictive models were highly similar, we provided a detailed description only for fluid intelligence for simplicity. Specifically, connections predicting higher fluid intelligence and younger age almost all located within networks, especially in VAN, DMN, and SMN. On the other hand, connections predicting older age and lower intelligence predominantly occurred between networks. This pattern was particularly evident between SMN and associative networks like VAN, FPN, and DAN, as well as between DMN and VAN (Figures 3C,4C). The network‐level representations of weight maps derived from models were similar whether we included all subjects, those with mean FD < 0.15, or those with FD < 0.20, were highly similar to those based on all subjects (Figure S2, Supporting Information).

### 2.4 Stability of Predictive Models

As shown in Figure 3C and Table S3, Supporting Information, the network weights had quite small standard deviations and 95% confidence interval. The inter‐correlations of weight maps across 2000 models ranged from *r*[force matching] = 0.9112 ± 0.0187 to *r*[*g*F] = 0.9760 ± 0.004 (Figure S4, Supporting Information). Further, predictive connectivity patterns derived from the bootstrap test and connectome‐based predictive modeling (CPM)^[^ ^23^, ^24^, ^25^, ^26^, ^27^ ^]^ were highly similar to those shown in Figure 3 (Figures S5,S6, Supporting Information). Moreover, the network‐level analysis revealed that none of the 36 network pairs achieved higher prediction accuracy than models based on the whole‐brain connections (Figure S7, Supporting Information). And the network size influences the prediction accuracy more than the network identity.

### 2.5 Association between Age, Cognition, and Functional Connectivity

**Figure 5:**
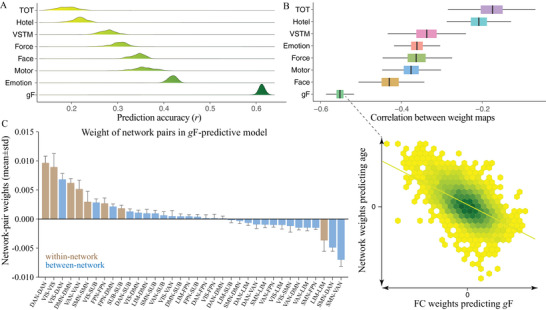
Prediction results of cognitive measures when controlling for the effect of age. A) Distribution of prediction accuracies across 200 repetitions of cross‐validation. B) Correlations of weight maps between age and each of the eight cognitive measures. Of all cognitive measures, fluid intelligence showed the highest similarity in weight maps with age. C) Mean weights distribution of within‐network and between‐network connections in *g*F‐predictive models. Error bars indicate standard deviation. DAN, dorsal attention network; DMN, default mode network; FPN, frontoparietal network; *g*F, fluid intelligence; LIM, limbic network; SMN, somatomotor network; SUB, subcortical network; VAN, ventral attention network; VIS, visual network.

After controlling for the effect of age, predictions remained significant for fluid intelligence, emotion expression recognition, force matching, and the hotel task (*p* < 0.01), but nonsignificant for the other measures. Moreover, when excluding age‐associated functional connections in model building, we observed comparable prediction accuracies and weight map similarities as achieved based on whole‐brain features (**Figure** **5**). Specifically, the strongest effect was observed between age and fluid intelligence, and greater strength of all within‐network connections (except the LIM) predicted higher fluid intelligence.

**Figure 6:**
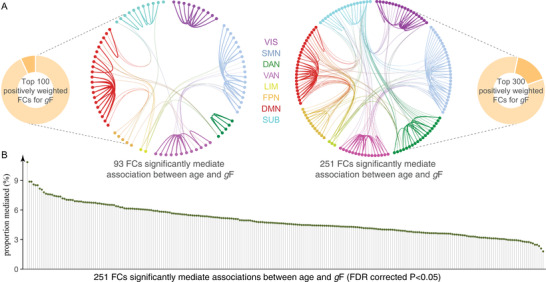
The *g*F‐predictive edges mediated the relationship between age and fluid intelligence. A) The indirect effect of age on fluid intelligence can be mediated by almost all *g*F‐predictive edges having the top 100 or top 300 positive weights. B) The proportion of mediated effect size ranged from 1.80% to 10.85%.

Among the top 100 connectivity edges having the highest positive predictive weights for fluid intelligence, 93 significantly mediated the relationship between age and fluid intelligence (FDR corrected *p* < 0.05, **Figure** **6**A). When expanding the mediation analysis to the top 300 positively weighted FCs, 251 showed a significant mediation effect (FDR corrected *p* < 0.05). The proportion of mediated effect size ranged from 1.80% to 10.85% (Figure 6B). However, the indirect effect of age on fluid intelligence cannot be significantly mediated by *g*F‐predictive edges with the highest negative weights (*p* > 0.05).

### 2.6 External Validation in Two Independent Datasets

**Figure 7:**
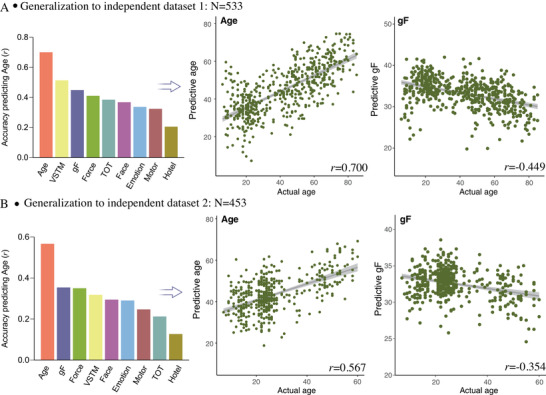
Generalization of predictive models in two external validation datasets. Predictive models were trained based on the full set of Cam‐CAN subjects for age and each of the eight cognitive metrics separately, and then directly applied to connectome features from each of the two independent datasets. Because the validation datasets did not include the corresponding cognitive measures as the Cam‐CAN, we calculated the correlation between model‐predicted cognitive scores and actual age. Accuracy is shown as the correlation between model‐predictive scores and actual age after controlling for mean framewise displacement. A) External dataset 1 includes 533 healthy subjects aged 6–85 years from NKI database. B) External dataset 2 includes 453 healthy subjects aged 7–60 years from Shanxi, China.

When applying the age‐predictive model defined in the full set of Cam‐CAN data to 533 healthy subjects from the NKI, we observe a significant correlation between actual and predicted age after covarying out the effect of mean FD (*r* = 0.70, *p* < 10^–16^, **Figure** **7**A). Meanwhile, when applying predictive models for each of the eight cognitive metrics to NKI subjects, the predicted cognitive scores are inversely correlated with age.

In the Shanxi dataset, the age‐predicted model trained in the Cam‐CAN data achieved a prediction accuracy of *r* = 0.567 (*p* < 10^–16^) between actual versus predicted age. Moreover, all models generalized to predict cognitive function for Shanxi data (*n* = 533), with the highest predictive performance reaching *r* = −0.354 (*p* < 10^–10^, Figure 7B) between predicted fluid intelligence scores and actual age after controlling for mean FD. All correlations survived the Bonferroni correction.

## 3 Discussion

In this study, we sought to examine the extent to which cognition and brain age overlap in the brain by using techniques from multivariate predictive modeling. Our first main finding is that individual difference in both cognitive function and age can be reliably predicted from an individual's unique patterns of brain connectivity and the models were generalizable across two external, heterogeneous datasets and robust to potential confounds. Our second main finding is that cognitive abilities shares common neural representations with brain age. Specifically, interrogation of overlapping brain patterns suggests that age‐related cognitive differences are associated with decreasing connectivity within networks (VAN, DMN, and SMN) and increasing connectivity between brain systems (SMN‐connected ones), reflecting a breakdown of the fine balance of network segregation and integration during aging (neural dedifferentiation).^[^ ^28^, ^29^ ^]^ Taken together, our findings offered new insights into the complex relationships among brain organization, cognition, and age.

Our present study was built on a series of brain imaging studies that have documented the large inter‐subject variability in how aging process relates to cognition and extended them in several ways. On one hand, we capitalized on advances in machine learning to achieve individual‐level estimation of cognitive scores and biological age in a purely data‐driven manner.^[^ ^12^, ^26^, ^30^ ^]^ The predictive modeling concerns more about aggregating the effects of thousands of age‐ or cognition‐associated variants into a single measure that best characterizes the aging brain, in light of the evidence that there may be unique aging trajectories with respect to distinct brain regions. The application of predictive analysis to whole‐brain functional connectivity may extend our knowledge about brain age prediction that is currently dominated by structural MRI studies.^[^ ^14^, ^17^ ^]^ On the other hand, the predictive signatures showed good generalizability across three independent datasets, indicating that our connectome‐based model captured the underlying brain‐behavior relationships that are sensitive to cognitive aging. These datasets were heterogeneous in several aspects in terms of sites, acquisition protocols, scanners, and sample characteristics. In this regard, the identified brain patterns provide new insights into cognitive aging research.^[^ ^31^, ^32^ ^]^

Evidence from existing studies has established that chronological age has a negative influence on individual's cognitive performance. Investigations aiming at revealing the neurobiological substrates of specific cognitive functioning often treat age as a covariate of no interest. A common practice is to regress it out in an effort to control for its potential effects on cognition.^[^ ^29^, ^33^, ^34^ ^]^ Instead of focusing on the independent influence of brain connectivity on cognition, our current study treated age and cognition as two separate biological processes, and pursued the approach of predictive neuroimaging^[^ ^23^ ^]^ to examine how they overlap in brain. Although these two constructs are strongly correlated, there are obvious differences in operationalization. The apparent correlation between age and cognition is a basic motivation that drives the current study to investigate the nature of their relationship by mapping behavioral associations to brain connections. In line with this, considering a high correlation between fluid intelligence and creativity, a recent study built a separate connectome‐based model for fluid intelligence and creativity and quantify the extent to which their predictive networks overlap in the brain.^[^ ^35^ ^]^ On the other hand, there is not a one‐to‐one mapping between age and cognition, but great individual differences exist in their association. Some older people can exhibit superior cognitive abilities than their age‐matched counterparts, and studies indicated that the brain network organization of those individuals was more similar to young adults.^[^ ^36^ ^]^ Further, growing evidence suggests that having an older‐appearing brain (with brain‐predicted age higher than the actual age) was associated with advanced cognitive decline.^[^ ^4^, ^5^, ^37^ ^]^ These findings indicate that cognitive functions may not be totally determined by age but follow the trajectory of brain changes. Moreover, independent lines of aging research suggest that there are intertwined interactions among age, cognition, and brain, and it is difficult to ascertain the precise relationship between any two, independent of the influence of the third variable.^[^ ^38^, ^39^, ^40^, ^41^ ^]^ For example, although we can factor out the influence of age in examining cognition‐brain associations, it will lead to limitations for subsequent interpretations. Since age explains a large variance of cognition, demonstrating significant variance explained by the brain beyond age can be difficult.^[^ ^38^ ^]^ Moreover, when predicting cognitive abilities with the effect of age factored out, what the model characterizes is the component of cognition that is independent of age, not allowing the interrogation of age‐accompanied neural substrates. Results shown in Figures 5,6 suggest that our cognition‐brain relationships are age‐invariant,^[^ ^29^ ^]^ and the predictive models captured neurobiological basis of cognition beyond what can be explained by age. Nevertheless, our results remain speculative and preliminary, and looking ahead, future study will be necessary to determine the robustness and replication of the current findings.

A primary finding of the current study is that age and cognition functions rely on overlapping functional systems in the brain. Specifically, weight maps of connectivity patterns extracted from the age‐predictive model demonstrated a highly negative correlation with those from the cognition‐predictive model. These results suggest that brain connectivity patterns stronger in older individuals are associated with greater cognitive impairment, while connections stronger in younger individuals are associated with higher cognitive abilities.^[^ ^24^ ^]^ Moreover, the validation analysis confirmed that the predictive models captured the brain patterns that were sensitive to the common components that are shared between age and cognition.^[^ ^35^, ^42^ ^]^

Regarding the above results, a natural hypothesis is that the age‐related decline in cognitive function may result from the effects of age on connectivity patterns.^[^ ^43^, ^44^, ^45^ ^]^ Our additional analysis demonstrated that functional connections positively predictive for fluid intelligence can significantly mediate the association between age and fluid intelligence. This finding is supported by previous studies showing that functional connectivity in specific networks is a relevant mediator of age‐related cognitive decline during normal aging.^[^ ^29^, ^43^, ^44^ ^]^ Our study adds to previous results suggesting that age may influence cognitive abilities indirectly through inducing changes in brain connectivity. However, this finding is preliminary and should be interpreted with caution, because a significant mediation effect is a measure of association that does not imply causal relationships. Nevertheless, these analyses represent a critical first step in characterizing associations between age, brain structure, and cognitive function that can be further explored in longitudinal studies.

Examination of the predictive models of age and cognitive decline revealed that within‐network connections exhibited negatively predictive power especially in VAN, DMN, and SMN; while between‐network connections, especially those connecting SMN and high‐order associative networks, played positive roles. This finding lend support to the theory that healthy cognitive aging is accompanied by decline in network segregation and enhancement in network integration, manifesting as decreasing functional connectivity within networks but increasing connectivity between networks.^[^ ^29^, ^44^ ^]^ Convergent findings suggest that a fine balance of segregated and integrated organization is a prerequisite for the brain systems to maintain functional specialization and efficient information processing.^[^ ^39^ ^]^

In this context, we speculated that the age‐associated loss of network segregation may be related to reduced ability and efficiency for specialized processing in the brain, which is consistent with the findings that reduced network segregation was closely related to decline in a variety of cognitive abilities including working memory, attention, and processing speed.^[^ ^42^ ^]^ At the same time, the effect of age‐related loss of network segregation also accords with the neural dedifferentiation hypothesis of aging, which is linked with impaired recruit of specialized brain regions during task performance, and an age‐related reduction in suppressing irrelevant network communications.^[^ ^46^, ^47^ ^]^ Notably, among all networks, the DMN and VAN demonstrated the great reductions of network segregation than others, which was in line with their established roles in cognitive aging. Specifically, weakened connectivity in DMN may influence its ability to shift from a task‐negative to task‐positive state, and further impair individual's cognitive abilities,^[^ ^18^ ^]^ supporting the hypothesis that DMN is the major site of pathology accumulation in multiple psychiatric disorders.^[^ ^48^ ^]^ Regarding VAN, considering its roles in detecting behaviorally relevant stimulus, and switching and coordinating dynamic transitions between DMN and FPN,^[^ ^25^ ^]^ decreases in network segregation of VAN may compromise its ability in allocating cognitive resources.^[^ ^49^, ^50^ ^]^ In contrast to a general decline in network segregation in other networks, the LIM network exhibited increasing within‐network connectivity. At first glance this might seem counterintuitive, however, growing evidence suggests that this reflects preserved emotional function and improved emotion regulation in older adults.^[^ ^51^, ^52^ ^]^ Moreover, this finding aligns with studies linking increased activation in hippocampal circuits to subsequent A*β* deposition and cognitive impairment,^[^ ^53^, ^54^, ^55^ ^]^ while targeting excess hippocampal activity benefited patients with amnestic mild cognitive impairment.^[^ ^56^ ^]^

Furthermore, the increased system integration, especially between SMN and associative networks, may suggest a compensational or over‐recruitment mechanism. This theory has gained support from independent lines of research that interpreted the age‐related shift toward higher network integration as an attempt to compensate for the decline of sensorimotor function in primary processing networks, to maintain cognitive performance as stable as possible.^[^ ^57^, ^58^ ^]^ In this respect, the increased connectivity between SMN and associative networks observed in the present study may imply an insufficient neural system,^[^ ^59^ ^]^ and a reflection of the over‐recruitment of high‐order cognitive networks to counteract behavioral decline due to reducing within‐network connections.^[^ ^60^ ^]^

The present study should be considered in light of some potential limitations. First, the current work was based on a cross‐sectional design, which may be influenced by cohort and period effects and not allowing us to draw any longitudinal inferences.^[^ ^39^, ^61^ ^]^ Investigations incorporating multiple time‐points of cognition and neuroimaging data are warranted to validate our results and make intra‐subject predictions of future cognitive developments.^[^ ^62^ ^]^ Second, the cognition‐ and age‐predictive models were developed based on linear models, therefore, they may not be sensitive to connectivity patterns following nonlinear trajectories along normal aging.^[^ ^63^, ^64^ ^]^ Third, despite a reduced complexity, using predictive modeling to reduce a rich set of connectivity features into a single estimate of brain age or cognition risks oversimplification, and thus may miss crucial patterns for understanding the underlying mechanisms.^[^ ^9^, ^24^ ^]^ Forth, separately building models for eight distinct cognitive measures using partial least square regression (PLSR) may be suboptimal, given that a prominent strength of PLSR lies in its ability to simultaneously predict multiple outcomes. It may therefore be interest to use PLSR as a multi‐output model to treat age and all eight cognitive metrics as a simultaneous outcome. As such, we would acquire a latent component representing functional connections that are involved in the age‐cognition interaction, as well as some age‐ or cognition‐specific components. Such a multi‐output model can be far more parsimonious and easier to interpret than having eight different models. Specifically, a recent fMRI study^[^ ^65^ ^]^ indicated that PLSR can achieved comparable accuracies in the setting of single‐ or multi‐output predictions, and more importantly, the most predictive features were highly overlapped (>90%) between these two types of models. Fifth, in model interpretation, we equated larger prediction weights with greater importance, which may risk over‐interpreting. On one hand, the backward models (methods for the decoding of neural information from data) may arbitrarily assign higher or lower weights to collinear features,^[^ ^66^ ^]^ because they may depend on noise components in the data, thereby making inferences difficult. On the other hand, the stability of feature weights can be low, and there exist asymmetric tendencies between seeking out‐of‐sample predictions and in‐sample inferences,^[^ ^67^ ^]^ especially when the sample size is small. Bzdok et al.^[^ ^67^ ^]^ also suggested that the identified important features were more likely to be consistent with each other between using linear models for prediction versus inferences when the sample size was >1000. Moreover, evidence from recent studies indicated that reproducible brain‐wide association studies require samples with thousands of individuals.^[^ ^68^ ^]^ Future studies should include as more participants as possible to capture reliable behavior‐associated brain patterns with large effect sizes. In light of these considerations, we show high stability of the feature weights and robust generalizability of predictive models across heterogenous datasets. Nevertheless, the interpretation and replication of the predictive models deserve further examination in future studies. Sixth, although a total of eight distinct cognitive measures were investigated, we focused more on fluid intelligence. This is mainly because fluid intelligence is a central cognitive measure, which reflects the general ability to solve novel reasoning problems.^[^ ^35^ ^]^ Moreover, fluid intelligence has large variability and is more predictable from brain connectivity than other cognitive measures, enabling the predictive modeling to adequately capture brain‐behavior associations.^[^ ^61^ ^]^ Further, although the weight maps showed high similarities across cognitive measures, it should be noted that the specific brain signatures for distinct cognitive metrics can differ significantly from each other. For example, in the prediction of emotion expression recognition and face recognition, connections within the LIM network showed the greatest contribution among all networks, which were less significant in the prediction of other cognitive measures. Specifically, in predicting motor learning, connections within the LIM network showed almost no predictive power. Within‐network connections in the SMN contributed the least to the prediction of visual short‐term memory (VSTM), but not for the other cognitive measures (Figure S5, Supporting Information). In this regard, the characterization of domain‐specific imaging biomarkers for each cognitive task merits further inquiry. Finally, in light of that fact the current analysis was performed on the basis of functional connectivity, which reflects the temporal dependence between neural activity across two distinct brain regions, we discussed the results mainly at the network level by highlighting which specific network pair has more predictive weight, rather than at the node level. Although the structural information may leak into functional data in the preprocessing, functional connectivity can provide unique insights that cannot be obtained from structural data, such as the inter‐network communications and brain dynamics.^[^ ^18^ ^]^ Our additional analyses indicated that grey matter volume achieved higher prediction accuracy than functional connectivity and integrating these two modalities further improved prediction (Table S4, Supporting Information). This result suggests that unique and complementary information encoded in distinct modalities can be used to better predict individual differences in phenotypes.^[^ ^69^ ^]^

## 4 Conclusions

In sum, we developed a functional connectivity‐based signature for brain age and cognitive aging, and demonstrated its generalizability across three independent datasets. Importantly, we revealed overlapping functional brain patterns for cognitive functions and brain age, which are characterized by decreased within‐network and increased between‐network connections. Overall, these findings provide direct evidence that cognitive aging is accompanied by disrupted network segregation and integration, reflecting a process of neural dedifferentiation and compensational theory during normal aging.

## 5 Experimental Section

### Discovery Dataset

Data used in the present study came from the Cambridge Centre for Ageing and Neuroscience (Cam‐CAN), which is a large‐scale, population‐based adult lifespan dataset aiming at uncovering neural underpinnings of cognitive aging.^[^ ^70^ ^]^ Participants included in this project were all cognitively healthy adults. Strict exclusion criteria were applied to recruit participants.^[^ ^71^ ^]^ Subjects with either missing age or unqualified fMRI data including degraded images and excessive head motion (described in MRI data acquisition section) were removed. Overall, 567 participants aged 18.5–88.9 years were retained for the main analyses (288 females, mean age 53.68 ± 18.37). Ethical approval for the Cam‐CAN study was obtained from the Cambridgeshire 2 (now East of England‐Cambridge Central) Research Ethics Committee, and written informed consent was obtained from each participant.

Participants completed a battery of behavioral tasks to assess their cognitive functions spanning domains of executive function, emotional processing, motor function, and memory. Specifically, the current study focused on eight cognitive tasks involving fluid intelligence task (Cattell Culture Fair), force matching, Hotel task, motor learning, tip‐of‐the‐tongue task (TOT), VSTM, face recognition (Benton test), and emotion expression recognition. A brief description for these behavioral tests can be found in Figure S8, Supporting Information. All test scores were standardized to *z*‐values. Cognitive metrics that were assessed using response times in seconds were reversed by multiplying by −1, so that a higher value always corresponds to better performance. In this study, fluid intelligence was adopted as the primary cognitive measure being investigated, for which complete data were available for 552 participants (277 females, mean age 53.81 ± 18.09). Included subjects for the other cognitive metrics varied from *n* = 263 (motor learning) to *n* = 556 (emotion expression recognition). As expected, all cognitive domain scores were negatively correlated with individual's age, reflecting a pattern of aging‐related cognitive decline (Bonferroni corrected *p* < 0.001, Figure S9, Supporting Information).

### External Dataset 1

FMRI scans from the enhanced Nathan Kline Institute (NKI)/Rockland Sample database, which is publicly available at the International Neuroimaging Data‐sharing Initiative (INDI) online were used as the independent validation dataset. This is a large‐scale, community‐ascertained lifespan sample with advanced neuroimaging and genetics.^[^ ^72^ ^]^ The study was approved by the NKI institutional review board and all subjects provided informed consent. In contrast to Cam‐CAN, NKI dataset had a wider age range spanning human development from childhood to late adulthood. In the current study, a total of 533 NKI subjects with complete fMRI scans aged 6–85 years were adopted as the validation data (338 females, mean age 42.35 ± 21.18).

### External Dataset 2

This independent dataset was locally collected through advertisements from the Department of Psychiatry at the First Hospital of Medical University, Shanxi, China. Similar inclusion and exclusion criteria were applied to include healthy subjects spanning human development from childhood to middle adulthood (age range 7–60 years).^[^ ^73^ ^]^ The study was approved by the Ethics Committee of the First Hospital of Shanxi Medical University and all participants provided written informed consent. Overall, a total of 453 subjects with complete resting‐state fMRI data aged of 26.86 ± 12.13 were included in the study (166 males, 287 females).

### MRI Data Acquisition

Details regarding MRI data acquisition and preprocessing can be found in Supporting Information. The data preprocessing strategy was the same as the previous publications,^[^ ^36^, ^74^ ^]^ and complied with the general framework in aging studies.^[^ ^75^, ^76^ ^]^

### Functional Parcellation and Connectome Construction

The Brainnetome atlas was utilized to delineate the brain into 246 macroscale regions of interest,^[^ ^77^ ^]^ serving as functional nodes. For each individual, the regional time series were generated by averaging voxel‐wise fMRI time series per node. Then, the pairwise Pearson correlations between all nodes’ time series were calculated and then Fisher z‐transformation was applied, yielding a 246 × 246 connectome matrix for each individual. Extracting the upper triangle elements of the matrix resulted in 30 135 unique edges for analyses.

### Development of Connectome‐Based Predictive Models

PLSR was employed to separately build predictive models for individual age and each of the eight cognitive metrics scores. Specifically, PLSR was capable of establishing reliable brain‐behavior relationships and was widely used in predictive neuroimaging.^[^ ^78^, ^79^ ^]^ Moreover, PLSR required no prior feature selection to achieve dimension reduction, as it worked by projecting high‐dimensional features into a small set of latent components,^[^ ^12^ ^]^ which could facilitate the comparison of predictive models across conditions.

The prediction analysis was placed in a tenfold CV framework to avoid circularity bias. Specifically, 90% of the data were designated as the training set, and the remaining 10% data were used as the testing set. Mode building was performed on training data, and the test data were kept independent of the training process to prevent any leakage between them. The predictive model learned from the training data was directly applied to testing set without any modification. Because the division of data folds was conducted randomly, shuffle‐split techniques were further employed by repeating the prediction procedure 200 times to control this influence. Model performance was quantified as the correlation *r* between actual and predicted scores, the CV *R* ^2^, as well RMSE, averaged across 200 repetitions.

### Predictive Network Anatomy and Overlap between Models

The relative contribution of each individual feature to prediction could be quantified by extracting the regression coefficients from the predictive model (the returned BETA in plsregress).^[^ ^80^, ^81^ ^]^ Averaging 2000 weight maps (200 repetitions × 10 folds), the connectivity‐level representation of the predictive model was generated. The node‐level interpretation was achieved by summarizing weight values of all connections incident to each of the 246 atlas‐defined functional macroscale regions.

To unveil networks playing a disproportionate role in explaining the success of predictive model, the whole‐brain nodes were first grouped into eight canonical networks defined by Brainnetome atlas including seven networks mapped from the Yeo's parcellation (visual, SMN, DAN, VAN, LIM, FPN, DMN), and the SUB.^[^ ^78^, ^82^ ^]^ Detailed information about the atlas and network definition can be found in Table S5, Supporting Information. Next, for each pair of networks, weights of all connections were added up, and then normalized them by the total number of connections belonging to that network pair to control for the influence of network size.^[^ ^27^ ^]^

The extent to which predictive models were similar to or distinct from each other was further evaluated. Specifically, the Pearson's correlation between averaged weight maps from the age‐predictive model and each of the eight cognition‐predictive models at the connection, node, and network level was calculated.^[^ ^82^ ^]^

### Examining the Stability of Predictive Models

A set of sensitivity analyses were performed to demonstrate the reliability of the predictive models. 1) The mean and standard deviation as well as 95% confidence interval of the network weights across 200 CV iterations are shown in all figures. 2) The stability of predictive weights was evaluated by calculating the inter‐correlations of weight maps across 2000 models. 3) A bootstrap test was performed to examine the stability of predictive weights. Specifically, bootstrap samples from the full data (random sampling of participants with replacement 5000 times) were iteratively generated, and a predictive model was built using each bootstrap sample. Then these weight maps were averaged to obtain a network‐level representation. 4) To confirm the predictive brain patterns were not influenced by multicollinearity, another widely‐used approach (CPM) was employed,^[^ ^23^, ^24^, ^25^, ^26^, ^27^ ^]^ to make predictions and identify predictive features. Detailed implementation can be found in the Supporting Information. 5) To further interrogate network contributions, the prediction framework was rerun using only within‐network or between‐network connections to predict age. A total of 36 distinct network pairs were tested.

### Evaluating Association between Age, Cognition, and Functional Connectivity

Following common practices conducted in aging research,^[^ ^38^, ^39^, ^40^, ^41^ ^]^ the following analyses were performed to show the cognition‐predictive models were not merely driven by age, instead, the cognition‐predictive FCs significantly mediated the association between age and fluid intelligence. 1) The prediction accuracies were calculated for all eight cognitive measures while controlling for the effect of age. 2) The effect of age on model building was controlled by excluding all age‐associated functional connections. Specifically, in the training dataset, the partial correlation between each FC and cognitive scores with age as a covariate was calculated, and only significantly correlated connections that were independent of the age effect were retained, which were further leveraged to make predictions. 3) The authors examined whether the cognition‐predictive connectivity patterns were able to mediate the association between age and fluid intelligence. In this case, age was used as an independent variable, fluid intelligence as the dependent variable, and each connectivity feature constituted a mediator. Gender and mean head motion were used as covariates.

### External Validation in Independent Datasets

The generalizability of the predictive models were further tested by examining whether model built in one dataset could be directly used to predict age or cognitive scores from connectivity data obtained from completely independent sites. Specifically, based on the full set of Cam‐CAN subjects, PLSR was first utilized to define an age‐predictive model using whole‐brain connectivity features. Next, the weight map from the constructed model was obtained by extracting the regression coefficient for each connectivity. Then, the dot product of vectorized whole‐brain connectivity patterns from each of these two validation datasets was calculated with the obtained weight map,^[^ ^82^, ^83^, ^84^ ^]^ yielding a model‐predicted age for each subject. A separate cognition‐predictive model for each of the eight cognitive metrics was also defined and it was applied to the validation cohorts. Because the validation datasets did not include the corresponding cognitive measures as the Cam‐CAN, the correlation between model‐predicted cognitive scores and actual age was calculated.

### Statistical Analysis

All data were expressed as the mean ± standard deviation. SIMPLS algorithm was used to solve PLSR as implemented in MATLAB R2016a (plsregress function). Significance of prediction accuracy was determined by permutation test (5000 iterations). Significance of similarity between weight maps was assessed using permutation test by permuting the weight maps 10 000 times. The significance of the indirect effects was assessed based on 10 000 bootstrap iterations, which were implemented in R (version 4.1.3) using the “mediation” package (version 4.5.0).

## Conflict of Interest

The authors declare no conflict of interest.

## Author Contributions

R.J., V.C., and J.S. conceived and designed the experiment. R.J. performed the analyses with support from N.Z., S.Q., and D.S., and J.W. provided guidance on result interpretation. R.J. and V.C. wrote the paper with contributions from J.S., D.S., and comments from all other authors.

## Supporting information

Supporting Information

## Data Availability

The data that support the findings of this study are available from the corresponding author upon reasonable request.
