# Supplementary material for: A Neuroimaging Signature of Cognitive Aging from Whole‐Brain Functional Connectivity
Source: Adv Sci (Weinh). 2022 Jul 10;9(24):2201621. doi: 10.1002/advs.202201621 (PMC9403648; doi:10.1002/advs.202201621)
Supplement: Supplementary file 1 — Supporting Information [file ADVS-9-2201621-s001.pdf]

ADVANCED SCIENCE
Open Access

Supporting Information

for *Adv. Sci.*, DOI 10.1002/advs.202201621

A Neuroimaging Signature of Cognitive Aging from Whole-Brain Functional Connectivity

Rongtao Jiang, Dustin Scheinost, Nianming Zuo, Jing Wu, Shile Qi, Qinghao Liang, Dongmei Zhi, Na Luo, Young-Chul Chung, Sha Liu, Yong Xu, Jing Sui* and Vince Calhoun*

Supporting Information

A neuroimaging signature of cognitive aging from whole-brain functional connectivity

Rongtao Jiang*[1], Dustin Scheinost[1,2,3,4], Nianming Zuo[5,6], Jing Wu[7], Shile Qi[8], Qinghao Liang[9], Dongmei Zhi[10], Na Luo[5,6], Young-Chul Chung[11,12], Sha Liu[13], Yong Xu[13], Jing Sui*[10], Vince Calhoun[14]

[1] Department of Radiology and Biomedical Imaging, Yale School of Medicine, New Haven, CT, USA, 06520
[2] Interdepartmental Neuroscience Program, Yale University, New Haven, CT 06520, USA
[3] Department of Statistics & Data Science, Yale University, New Haven, CT 06520, USA
[4] Child Study Center, Yale School of Medicine, New Haven, CT 06510, USA
[5] Brainnetome Center and National Laboratory of Pattern Recognition, Institute of Automation, Chinese Academy of Sciences, Beijing, China, 100190
[6] School of Artificial Intelligence, University of Chinese Academy of Sciences, Beijing, China, 100049
[7] Department of Medical Oncology, Beijing You-An Hospital, Capital Medical University, Beijing, China, 100069
[8] College of Computer Science and Technology, Nanjing University of Aeronautics and Astronautics, Nanjing, China, 211106
[9] Department of Biomedical Engineering, Yale University, New Haven, CT, USA, 06520
[10] State Key Laboratory of Cognitive Neuroscience and Learning, Beijing Normal University, Beijing, China, 100088
[11] Department of Psychiatry, Jeonbuk National University Medical School, Jeonju, Republic of Korea, 54907
[12] Department of Psychiatry, Chonbuk National University Hospital, Jeonju, Republic of Korea 54907
[13] Department of Psychiatry and MDT Center for Cognitive Impairment and Sleep Disorders, First Hospital, First Clinical Medical College of Shanxi Medical University, Taiyuan, China, 030001
[14] Tri-institutional Center for Translational Research in Neuroimaging and Data Science (TReNDS), Georgia Institute of Technology, Emory University and Georgia State University, Atlanta, GA, USA, 30303

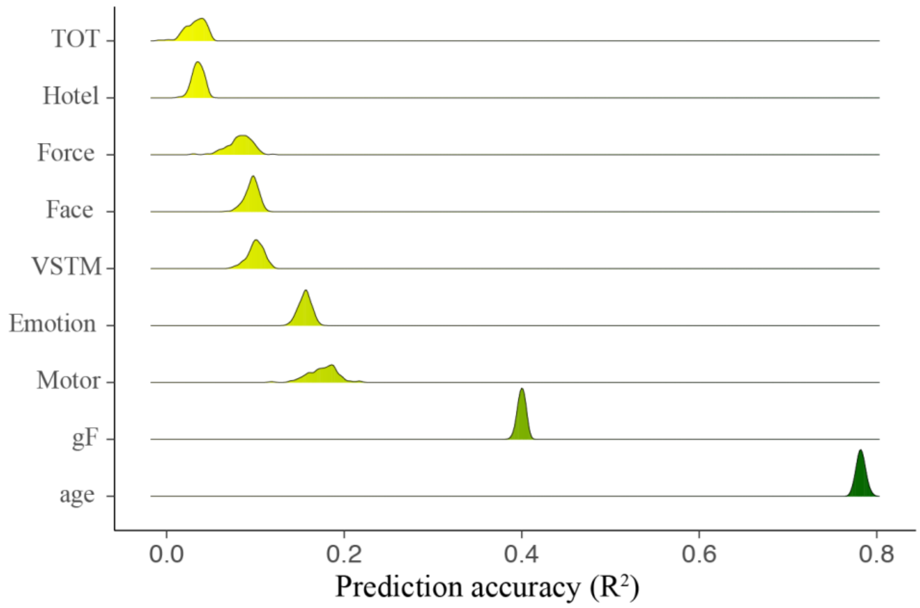

Figure S1. Distribution of the fraction of explained variance (R^2) across 200 repetitions of cross-validation for age and each of the eight cognitive metrics.

Table S1. Prediction accuracies of age and cognitive metrics, and influence of covariates

	r	***RMSE***	***Control for age***	***Control for gender***	***Control for FD***
**Age**	0.885±0.0028	8.569±0.0964	NA	0.885±0.0028	0.841±0.0039
**Emotion expression**	0.422±0.0060	9.860±0.041	0.205±0.0091	0.417±0.0060	0.373±0.0076
**Face recognition**	0.361±0.0066	2.26±0.0093	0.029±0.0097^{NS}	0.359±0.0066	0.281±0.0081
**Fluid intelligence**	0.634±0.0029	5.165±0.017	0.253±0.0054	0.634±0.0029	0.528±0.0040
**Force matching**	0.333±0.0194	0.040±5.0e-4	0.149±0.0228	0.329±0.0194	0.263±0.0210
**Hotel task**	0.250±0.0077	166.55±0.58	0.119±0.0101	0.250±0.0077	0.183±0.0093
**Motor learning**	0.441±0.0134	0.035±3.6e-4	0.081±0.0218^{NS}	0.440±0.0134	0.332±0.0164
**Tip-of-tongue**	0.254±0.0104	0.240±0.001	0.047±0.0139^{NS}	0.254±0.0104	0.212±0.0117
**VSTM**	0.366±0.0080	0.758±1.0e-4	0.026±0.0117^{NS}	0.359±0.0078	0.306±0.0094

FD, framewise displacement; NA, not applicable; NS, nonsignificant; RMSE, root mean square error; VSTM, visual short-term memory.

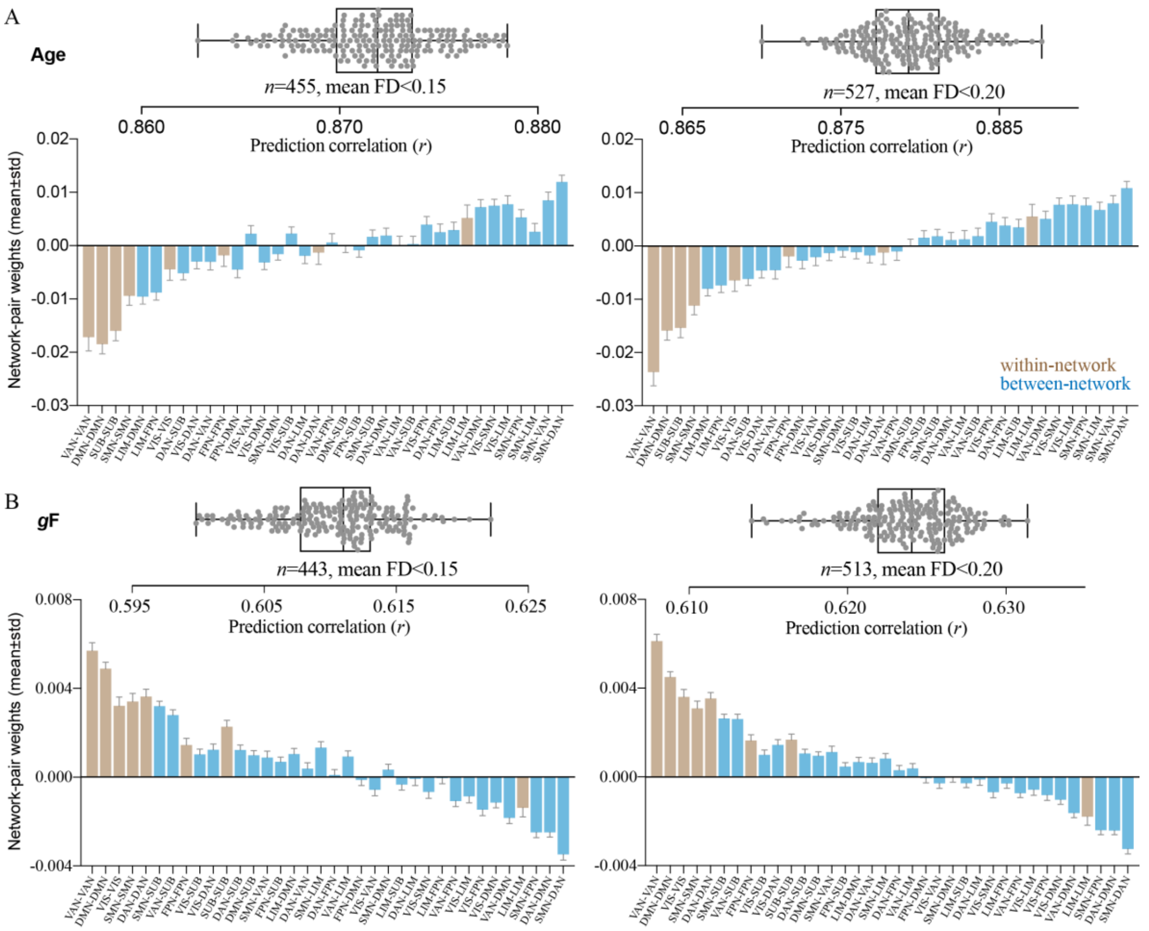

Figure S2. Prediction correlations remain largely unchanged when only including subjects with a mean framewise displacement (FD) <0.15 or FD <0.20, suggesting that the predictive models are robust to head motion. The network-level representations of weight maps derived from models build on subjects with mean FD <0.15, and subjects with mean FD <0.20, were highly similar to those based on all subjects. For better comparison, network pairs in the bar plot were shown by the same sequence as in Figure 3C. Subplot A shows results for age-predictive models, and subplot B shows results for *g*F-predictive models.

Table S2. Top 10 weighted functional nodes in predicting age or fluid intelligence

Age-predictive model				gF-predictive model		
Top 10 nodes with the highest positive weights in predicting age and negative weights in predicting gF						
Rank	Weights	Region	MNI	Weights	Region	MNI
#1	0.02999	R. hippocampus	(22,-12,-20)	-0.00450	R. rostral parahippocampal gyrus	(28,-8,-33)
#2	0.02869	L. occipital polar cortex	(-18,-99,2)	-0.00418	R. entorhinal cortex	(19,-10,-30)
#3	0.02422	R. dorsolateral superior frontal gyrus	(20,4,64)	-0.00378	R. parahippocalpal gyrus TI	(22,1,-36)
#4	0.02416	R. parahippocampal gyrus	(19,-36,-11)	-0.00349	L. entorhinal cortex	(-19,-12,-30)
#5	0.02382	L. superior temporal gyrus	(-50,-11,1)	-0.00308	L. pre-motor thalamus	(-18,-13,3)
#6	0.02189	L. inferior frontal gyrus	(-39,23,4)	-0.00305	R. medial precuneus	(6,-65,51)
#7	0.02119	R. precuneus	(16,-64,25)	-0.00303	L. precuneus (dmPOS)	(-12,-67,25)
#8	0.02049	L. caudoposterior superior temporal sulcus	(-52,-50,11)	-0.00302	L. parahippocalpal gyrus TI	(-23,2,-32)
#9	0.01970	L. rostroposterior superior temporal sulcus	(-54,-40,4)	-0.00297	R. precuneus (dmPOS)	(16,-64,25)
#10	0.01860	R. middle frontal gyrus	(42,27,39)	-0.00297	R. hippocampus	(22,-12,-20)
Top 10 nodes with the highest negative weights in predicting age and positive weights in predicting gF						
#1	-0.03344	R. caudate cingulate gyrus	(6,-20,40)	0.00872	R. caudal temporal thalamus	(10,-14,14)
#2	-0.03196	R. caudal temporal thalamus	(10,-14,14)	0.00729	R. dorsal caudate	(14,5,14)
#3	-0.02817	R. inferior frontal gyrus	(54,24,12)	0.00656	L. dorsal caudate	(-14,2,16)
#4	-0.02548	R. posterior parietal thalamus	(15,-25,6)	0.00617	L. ventral caudate	(-12,14,0)
#5	-0.02532	L. lingual gyrus	(-17,-60,-6)	0.00600	L. caudal temporal thalamus	(-12,-22,13)
#6	-0.02319	L. precentral gyrus	(-32,-9,58)	0.00547	R. posterior parietal thalamus	(15,-25,6)
#7	-0.02261	L. ventral caudate	(-12,14,0)	0.00513	R. superior temporal gyrus	(47,12,-20)
#8	-0.01976	R. medial orbital gyrus	(6,57,-16)	0.00508	R. insular gyrus	(39,-2,-9)
#9	-0.01881	R. dorsal caudate	(14,5,14)	0.00486	L. rostroventral cingulate gyrus	(-3,8,25)
#10	-0.03344	R. insular gyrus	(39,-2,-9)	0.00475	R. hypergranular insula	(37,-18,8)

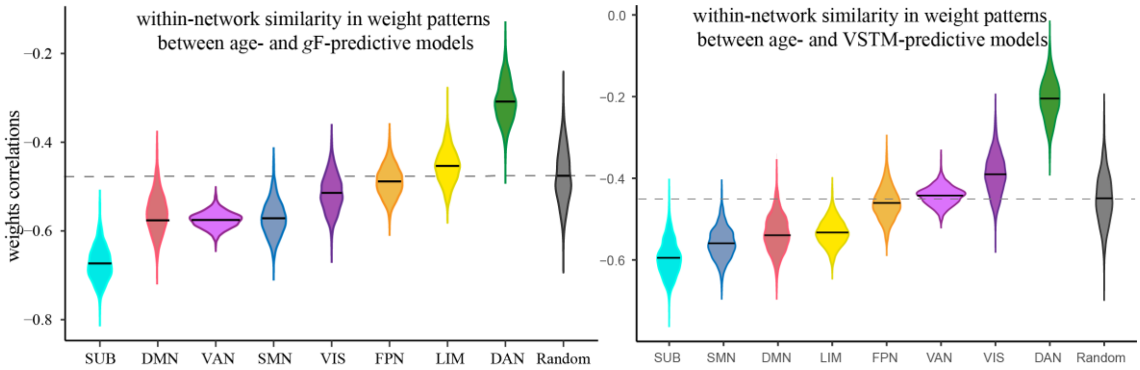

Figure S3. Similarity of within-network weight maps between age- and cognition-predictive models. To examine whether within-network weight maps between age- and cognition-predictive models show higher similarities than randomly selected connections, we conducted a bootstrap test. Specifically, for each functional network we randomly selected 200 within-network connections without replacement 1000 times; and then calculated the correlation of weight maps from age-predictive and cognition-predictive models for each iteration. Further, we randomly selected 200 connections from the whole connectome 1000 times and calculated correlations of weight maps from age-predictive and cognition-predictive models for each iteration. Differences between the within-network weight maps and randomly selected weight maps were compared using a two-sample t-test. Overall, among all eight networks, only DAN and LIM have lower similarity in weight patterns than a matched number of randomly selected connections. For the VSTM-predictive model, DAN and VIS have lower similarity in weight patterns than a matched number of randomly selected connections.

Table S3. Predictive weights and 95% confidence interval for each network pair

	Age		Fluid intelligence	
	**Mean**	**95% CI**	**Mean**	**95% CI**
VIS-VIS	-0.0070	[-0.00707, -0.00689]	0.0036	[0.00355, 0.00357]
VIS-SMN	0.0062	[0.00613, 0.00624]	-0.0003	[-0.00026, -0.00024]
SMN-SMN	-0.0127	[-0.01273, -0.01258]	0.0034	[0.00336, 0.00339]
VIS-DAN	-0.0052	[-0.00525, -0.00514]	0.0013	[0.00133, 0.00135]
SMN-DAN	0.0115	[0.01149, 0.0116]	-0.0032	[-0.00323, -0.00321]
DAN-DAN	-0.0002	[-0.00032, -0.00013]	0.0034	[0.00334, 0.00337]
VIS-VAN	-0.0020	[-0.00207, -0.00194]	-0.0002	[-0.00016, -0.00014]
SMN-VAN	0.0097	[0.0096, 0.00973]	0.0010	[0.00099, 0.00101]
DAN-VAN	-0.0043	[-0.00432, -0.00419]	0.0006	[0.00058, 0.0006]
VAN-VAN	-0.0224	[-0.02249, -0.02226]	0.0056	[0.00561, 0.00563]
VIS-LIM	0.0071	[0.00699, 0.00712]	-0.0007	[-0.00073, -0.00071]
SMN-LIM	0.0072	[0.00717, 0.0073]	0.0006	[0.00057, 0.00059]
DAN-LIM	-0.0007	[-0.00076, -0.00064]	-0.0002	[-0.00022, -0.0002]
VAN-LIM	0.0019	[0.00186, 0.002]	0.0002	[0.00022, 0.00024]
LIM-LIM	0.0045	[0.00437, 0.00457]	-0.0020	[-0.00206, -0.00203]
VIS-FPN	0.0038	[0.00377, 0.0039]	-0.0008	[-0.00085, -0.00083]
SMN-FPN	0.0072	[0.00713, 0.00725]	-0.0024	[-0.0024, -0.00238]
DAN-FPN	0.0039	[0.00381, 0.00394]	0.0004	[0.00038, 0.00039]
VAN-FPN	-0.0004	[-0.00043, -0.00028]	-0.0007	[-0.00069, -0.00067]
LIM-FPN	-0.0076	[-0.00763, -0.00752]	-0.0003	[-0.00029, -0.00028]
FPN-FPN	-0.0028	[-0.00293, -0.00275]	0.0018	[0.0018, 0.00182]
VIS-DMN	-0.0018	[-0.00188, -0.00176]	-0.0012	[-0.00117, -0.00116]
SMN-DMN	-0.0018	[-0.00182, -0.00172]	-0.0001	[-0.00016, -0.00014]
DAN-DMN	0.0017	[0.00165, 0.00176]	-0.0025	[-0.00255, -0.00254]
VAN-DMN	0.0060	[0.00592, 0.00604]	-0.0018	[-0.00185, -0.00183]
LIM-DMN	-0.0078	[-0.00781, -0.0077]	0.0007	[0.00064, 0.00066]
FPN-DMN	-0.0028	[-0.00283, -0.00271]	0.0001	[0.0001, 0.00012]
DMN-DMN	-0.0167	[-0.0168, -0.01664]	0.0047	[0.00468, 0.0047]
VIS-SUB	-0.0013	[-0.00135, -0.00124]	0.0013	[0.00134, 0.00136]
SMN-SUB	0.0011	[0.00104, 0.00115]	0.0026	[0.00256, 0.00257]
DAN-SUB	-0.0062	[-0.00623, -0.00612]	0.0012	[0.00116, 0.00117]
VAN-SUB	0.0022	[0.00214, 0.00226]	0.0024	[0.00243, 0.00245]
LIM-SUB	0.0038	[0.00377, 0.0039]	-0.0002	[-0.00023, -0.00021]
FPN-SUB	0.0008	[0.0007, 0.00081]	0.0007	[0.00072, 0.00074]
DMN-SUB	0.0006	[0.00052, 0.00062]	0.0012	[0.00115, 0.00117]
SUB-SUB	-0.0132	[-0.01326, -0.0131]	0.0012	[0.00118, 0.0012]

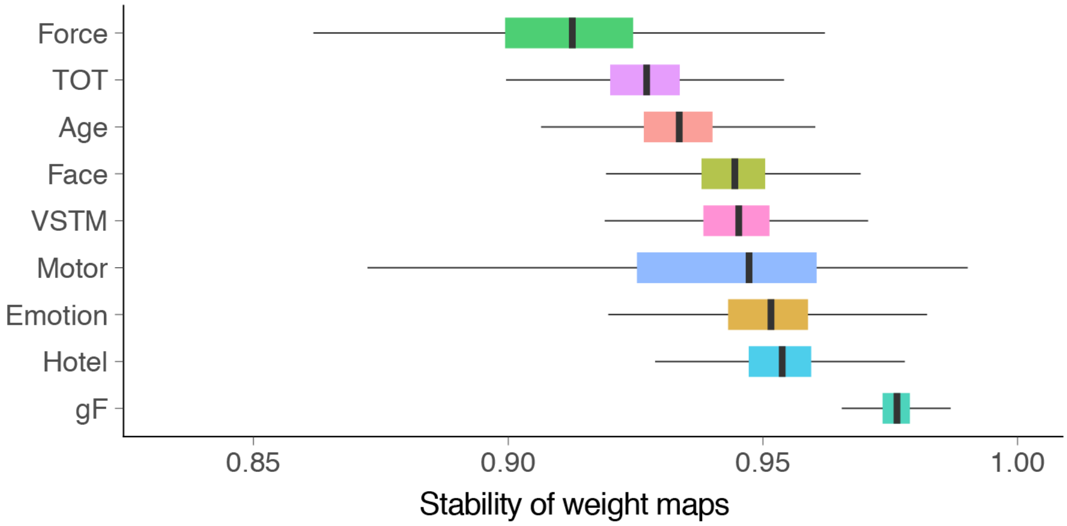

Figure S4. Stability of weight maps across 2000 distinct models. The prediction analysis was placed within a 10-fold cross-validation with 200 repetitions, generating 2000 predictive models in total. Stability of the predictive models was evaluated by calculating the inter-correlations of weight maps across 2000 models. DAN, dorsal attention network; DMN, default mode network; FPN, frontoparietal network; gF, fluid intelligence; LIM, limbic network; SMN, somatomotor network; SUB, subcortical network; VAN, ventral attention network; VIS, visual network; VSTM, visual short-term memory.

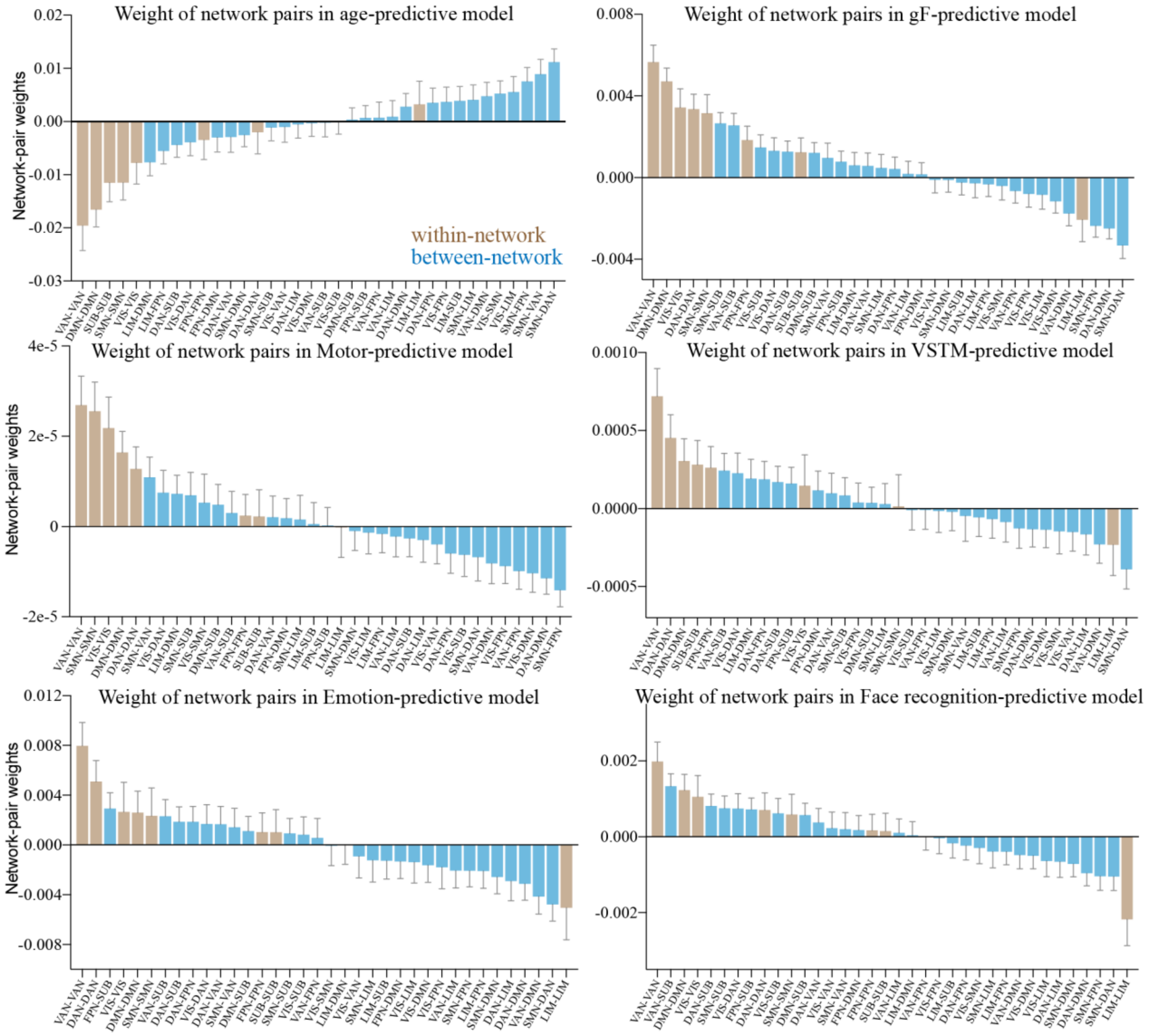

Figure S5. Mean weights distribution of within-network and between-network connections derived from bootstrap tests. We iteratively generated bootstrap samples by randomly sampling participants with replacement (5000 iterations), and then built predictive models using each bootstrap sample. Error bars indicate standard deviation. DAN, dorsal attention network; DMN, default mode network; FPN, frontoparietal network; gF, fluid intelligence; LIM, limbic network; SMN, somatomotor network; SUB, subcortical network; VAN, ventral attention network; VIS, visual network; VSTM, visual short-term memory.

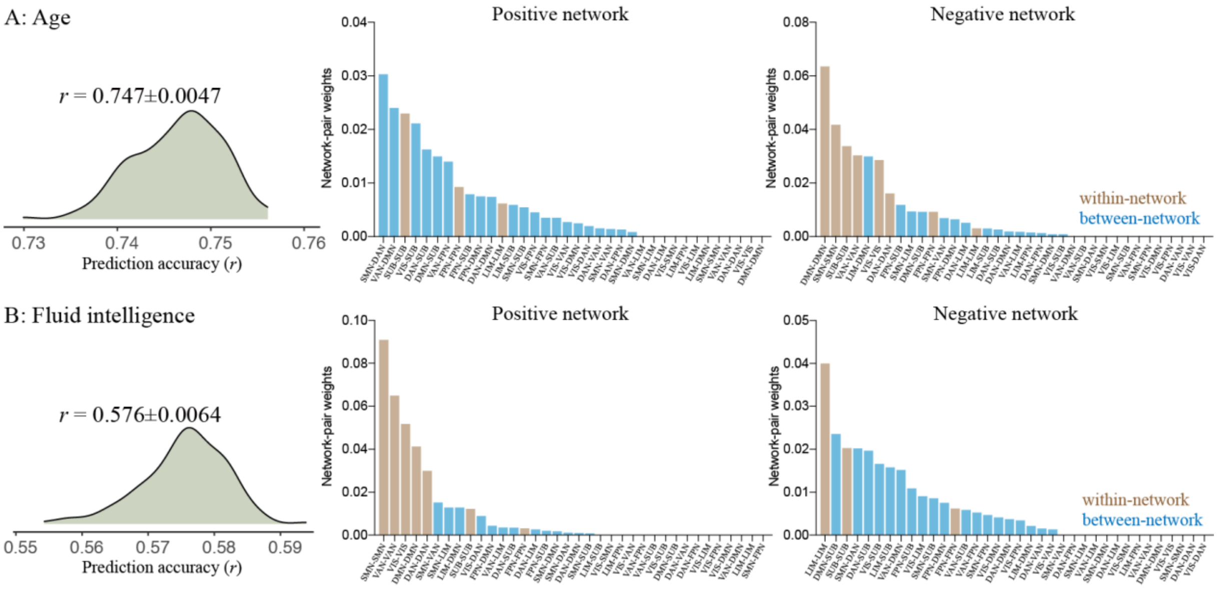

Figure S6. Predictive results based on connectome-based predictive modeling (CPM) [1-5], CPM works by (i) calculating the correlation of each connection to the target measure (e.g., age) across training subjects, and retaining the most significantly correlated ones under a predefined threshold; (ii) separating the selected features into a positive tail (the positively-correlated connections) and a negative tail (the negatively-correlated connections); (iii) separately summing the selected connections in the positive and negative tails into a single aggregate metric (positive network strength, negative network strength); (iv) submitting the aggregate metrics to a linear regression model. Detailed implementation can be found in [1, 2]. Overall, results showed that the CPM method achieved slightly lower prediction accuracy than PLSR, but the identified predictive patterns were highly similar to those revealed by PLSR.

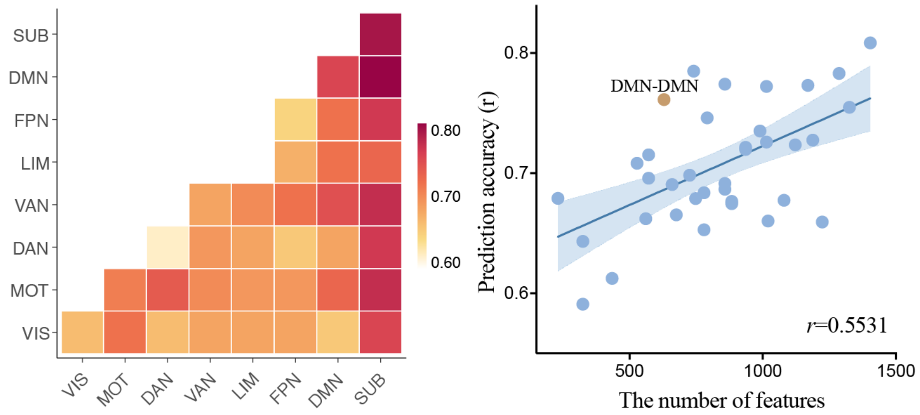

Figure S7. Prediction accuracies based on within- or between-network connections. To examine which functional network contributes more to the prediction than others, we reran the prediction framework using only within-network or between-network connections to predict age. All network pairs achieved a prediction accuracy lower than models based on whole-brain features. However, we found that networks having more connections are more likely to better predict age. Nevertheless, there are some interesting findings. For example, there are only a medium number of connections within DMN. But it achieved a relative higher accuracy in predicting age than its size-matched counterparts.

Table S4. Prediction results based on multimodal neuroimaging features

	Age	*Fluid intelligence*	
		No control for age	*Control for age*
**Functional connectivity**	0.885±0.0028	0.634±0.0029	0.253±0.0054
**Grey matter volume**	0.902±0.0021	0.640±0.0059	0.264±0.0058
**FCs+GMV**	0.932±0.0017	0.692±0.0034	0.326±0.0078

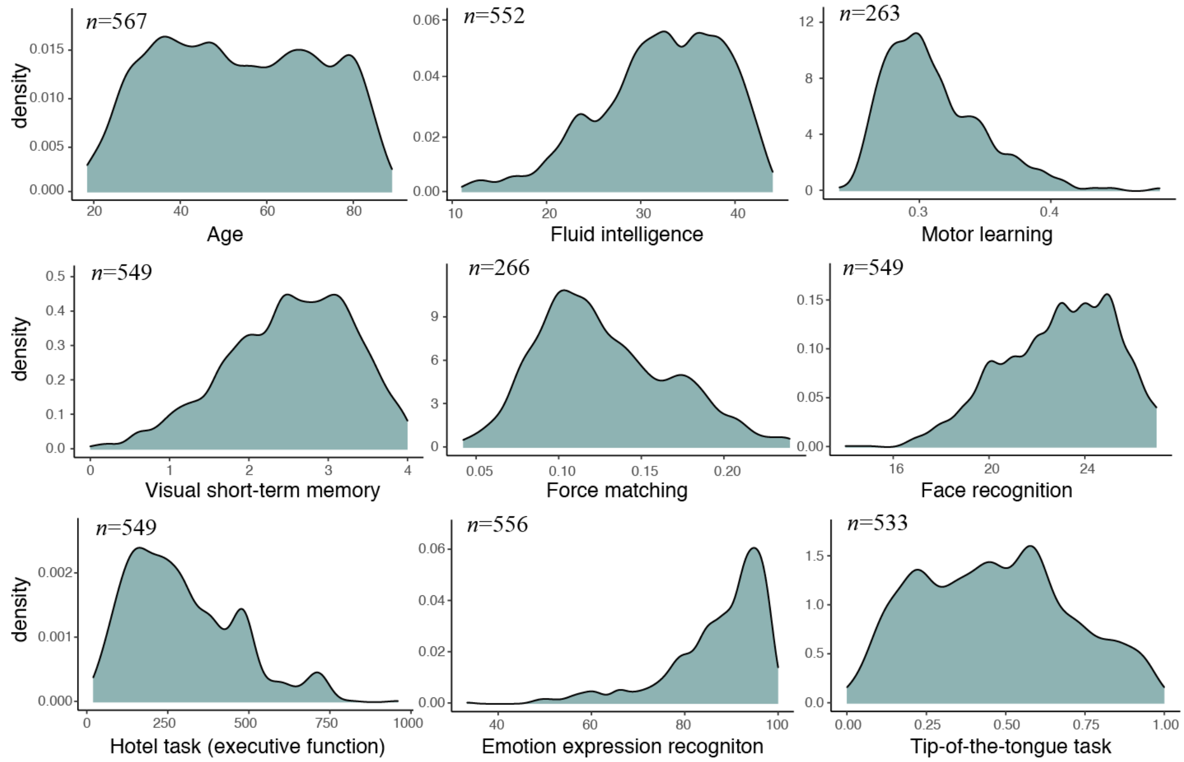

Figure S8. Distributions of age and each of the eight cognitive metrics scores. For most of the cognitive tasks, there were a comparable number of subjects, while for force matching and motor learning, the number of participants was reduced by half. This is mainly because most of the cognitive measures were derived from a paper-and-pencil task or simple computerized experiment. However, the motor learning and force matching required specialist equipment. To facilitate the efficiency of data acquisition, the cognitive measures were collected from 4 Cam-Can sessions. Specifically, all participants attended Session 1 and Session 2, and either Session 3a or Session 3b [6]. Cognitive measures of force matching and motor learning only appeared in either Session 3a or Session 3b. Therefore, only half of the participants have available data for these two cognitive tasks. Description for each of the eight behavioral tasks were directly copied from [7], while more details can be found in [6].

1) Fluid intelligence: Fluid intelligence was assessed using the standard form of the Cattell Culture Fair, Scale 2 Form A. Participants completed nonverbal puzzles involving series completion, classification, matrices, and conditions. Correct responses are given a score of 1 for a total maximum score of 46.
2) Motor learning: This task taps into motor adaptation, the process of learning new kinematic control in response to deviations in a voluntary action. Time-pressured movement of a cursor to a target by moving an (occluded) stylus under veridical, perturbed (30°), and reset (veridical again) mappings between visual and real space.
3) Visual short-term memory: View (1–4) colored discs briefly presented on a computer

screen, then after a delay, attempt to remember the color of the disc that was at a cued location, with response indicated by selecting the color on a color wheel (touchscreen input).

4) Force matching: Match mechanical force applied to left index finger by using right index finger either directly, pressing a lever which transmits force to left index finger, or indirectly, by moving a slider which adjusts the force transmitted to the left index finger. Accuracy was assessed by average difference between target force and matched force applied by participant via (direct, indirect) means.
5) Face recognition: Given a target image of a face, identify same individual in an array of 6 face images (with possible changes in head orientation and lighting between target and same face in the test array).
6) Hotel task: This task examines aspects of executive function that are important for complex planning and multitasking. Perform tasks in role of hotel manager: write customer bills, sort money, proofread advert, sort playing cards, alphabetise list of names. Total time must be allocated equally between tasks; there is not enough time to complete any one task.
7) Emotion expression recognition: View face and label emotion expressed (happy, sad, anger, fear, disgust, surprise) where faces are morphs along axes between emotional expressions.
8) Tip-of-tongue task: View faces of famous people (actors, musicians, politicians, etc.) and respond with the person's name, or “don't know” if they do not know the person's name (even if familiar), or “TOT” if they know the person's name but are (temporarily) unable to retrieve it.

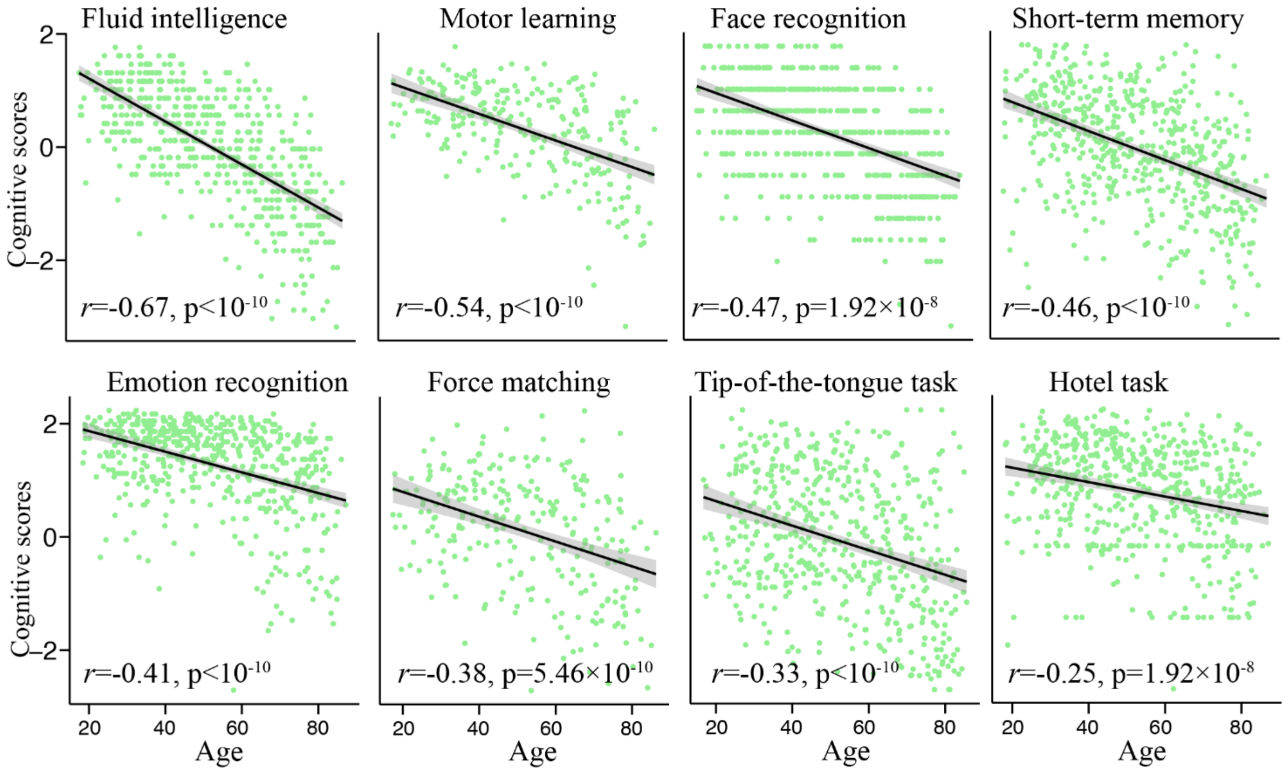

Figure S9. Correlations between cognitive metrics and age. As expected, all cognitive domain scores were negatively correlated with individual's age, reflecting a pattern of aging-related cognitive decline (p<0.001, Bonferroni corrected).

MRI data acquisition

Cam-CAN: Details of fMRI data acquisition can be found in [6, 7]. Briefly, resting-state scans were collected while participants rested with their eyes closed. In the movie-watching task, participants were scanned while they watched an excerpt of a compelling but unfamiliar film: "Bang! You're Dead", which is condensed from its original time of about 30 min to 8 min with the essential plot preserved. In the sensorimotor task, participants respond to 129 trials consisting of an initial practice trial, 120 bimodal audio/visual trials, and eight unimodal trials included to discourage strategic responding to one modality.

Imaging data were acquired using a 3T Siemens TIM Trio scanner with a 32-channel head coil. A 3D structural MRI was performed on each participant using a T1-weighted sequence with generalized autocalibrating partially parallel acquisition acceleration factor 2; repetition time (TR) = 2250 ms; echo time (TE) = 2.99 ms; flip angle = 9°; field-of-view (FOV) = 256 × 240 × 192 mm; resolution = 1 mm. For resting-state and sensorimotor task fMRI acquisition, T2*-weighted gradient echo planar image (EPI) data of 261 volumes were acquired with 32 slices (descending order) of thickness 3.7 mm and a slice gap of 20% for whole-brain coverage (TR = 1970 ms; TE = 30 ms; flip angle = 78°; FOV = 192 × 192 mm; resolution = 3 × 3 × 4.44 mm). Imaging data during the movie-watching task were acquired using a multi-echo EPI scan

with the following parameters: TR = 2470 ms; 5 echoes (TE = 9.4 ms, 21.2 ms, 33 ms, 45 ms, 57 ms); flip angle = 78°; FOV = 192 × 192 mm; resolution = 3 × 3 × 4.44 mm; slices = 32; 193 volumes.

NKI: Imaging data were acquired using a 3T Siemens TIM Trio scanner. Resting fMRI data were acquired using an EPI sequence with the following parameters: TR= 2500 ms; TE = 30 ms; flip angle = 80°; FOV = 216 mm; slice thickness = 3.0 mm, slices = 38, voxel size = 3.0 × 3.0 × 3.0 mm, acquisition time=5 minutes. High resolution T1 MPRAGE anatomical images were acquired with the following parameters: TR = 1900 ms, TE = 2.52 ms, slice thickness = 1.0 mm, flip angle = 9°, FOV= 256 mm, and voxel size = 1.0 × 1.0 × 1.0 mm.

Shanxi: MRI data were obtained with a Siemens Trio 3.0 Tesla scanner (Erlangen, Germany). Participants were instructed to stay awake with their eyes closed, and not to fall asleep or move during scanning. No participants were excluded due to falling asleep or opening their eyes. Functional scans were collected using an EPI sequence with the following parameters: TR = 2500 ms; TE = 30 ms; flip angle = 90°; FOV = 240 × 240 mm; slice thickness = 3 mm, slices=32; voxel size = 3.75 × 3.75 × 4 mm, 212 volumes.

Preprocessing

The DiffusionKit (diffusion.brainnetome.org) and in-house code were used for fMRI preprocessing, following the general framework in aging studies [8, 9]. We applied similar preprocessing strategy to all three datasets, which was the same as our previous publications. The BOLD echo planar image data for all three states were unwrapped based on field-map images to compensate for magnetic field inhomogeneities, realigned to correct motion effects where the motion parameters for each volume image were stored for the following regression, and slice-time corrected. The first 10 volumes were discarded to allow for magnetic equilibration and then nonlinearly registered to MNI 3-mm space (for validation datasets, we did not discard any volumes because they only included a small number of volumes). We further scrubbed the frames with excessive head motions based on framewise displacement (FD) >0.5 mm criterion and corrected the frames by interpolation. We discarded images with less than 40% of their original data after scrubbing. Moreover, fMRI scans with a mean FD>0.3 mm were excluded from further analysis. We then band-pass filtered the data at 0.009–0.08 Hz to reduce low-frequency drift and high-frequency noise. CompCor was used to reduce physiological effects as performed in [10, 11]. Specifically, the mean signal and 5 principal components of white matter and cerebrospinal fluid and movement parameters and their derivatives were regressed out as confounding factors to remove physiological noise. The aforementioned principal components were derived separately by decomposing the regional signal masked by the eroded

white matter and cerebrospinal fluid. In light of the fact that the location of functional regions was more variable in older adults, which can be alleviated by smoothing [11], we smoothed the volume images by a Gaussian filter with a kernel size of 6 mm. Considering a controversial physiological interpretation, global signal regression was not performed here. As previous studies confirmed the advantages of longer scan length, we concatenated fMRI time series from all three fMRI conditions [12-14]. Time courses from the task fMRI were calculated based on the raw task fMRI data, with no regression of task-evoked activity [15], resulting in a total length of 685 time points for Cam-CAN data. For validation cohorts, only resting-state fMRI was available, therefore, the total length of time points did not change.

Table S5. Network definition of the 246 brain nodes

	name	*region*	*Network*	*MNI*		*name*	*region*	*Network*	*MNI*
1	A8m	SFG_L_7_1	6	-5, 15, 54	124	cpSTS	pSTS_R_2_2	4	57, -40, 12
2	A8m	SFG_R_7_1	4	7, 16, 54	125	A7r	SPL_L_5_1	3	-16, -60, 63
3	A8dl	SFG_L_7_2	7	-18, 24, 53	126	A7r	SPL_R_5_1	3	19, -57, 65
4	A8dl	SFG_R_7_2	6	22, 26, 51	127	A7c	SPL_L_5_2	3	-15, -71, 52
5	A9l	SFG_L_7_3	7	-11, 49, 40	128	A7c	SPL_R_5_2	3	19, -69, 54
6	A9l	SFG_R_7_3	7	13, 48, 40	129	A5l	SPL_L_5_3	3	-33, -47, 50
7	A6dl	SFG_L_7_4	3	-18, -1, 65	130	A5l	SPL_R_5_3	3	35, -42, 54
8	A6dl	SFG_R_7_4	3	20, 4, 64	131	A7pc	SPL_L_5_4	2	-22, -47, 65
9	A6m	SFG_L_7_5	2	-6, -5, 58	132	A7pc	SPL_R_5_4	2	23, -43, 67
10	A6m	SFG_R_7_5	2	7, -4, 60	133	A7ip	SPL_L_5_5	3	-27, -59, 54
11	A9m	SFG_L_7_6	7	-5, 36, 38	134	A7ip	SPL_R_5_5	3	31, -54, 53
12	A9m	SFG_R_7_6	6	6, 38, 35	135	A39c	IPL_L_6_1	1	-34, -80, 29
13	A10m	SFG_L_7_7	7	-8, 56, 15	136	A39c	IPL_R_6_1	1	45, -71, 20
14	A10m	SFG_R_7_7	7	8, 58, 13	137	A39rd	IPL_L_6_2	6	-38, -61, 46
15	A9/46d	MFG_L_7_1	4	-27, 43, 31	138	A39rd	IPL_R_6_2	6	39, -65, 44
16	A9/46d	MFG_R_7_1	6	30, 37, 36	139	A40rd	IPL_L_6_3	3	-51, -33, 42
17	IFJ	MFG_L_7_2	6	-42, 13, 36	140	A40rd	IPL_R_6_3	3	47, -35, 45
18	IFJ	MFG_R_7_2	6	42, 11, 39	141	A40c	IPL_L_6_4	7	-56, -49, 38
19	A46	MFG_L_7_3	6	-28, 56, 12	142	A40c	IPL_R_6_4	6	57, -44, 38
20	A46	MFG_R_7_3	6	28, 55, 17	143	A39rv	IPL_L_6_5	3	-47, -65, 26
21	A9/46v	MFG_L_7_4	6	-41, 41, 16	144	A39rv	IPL_R_6_5	7	53, -54, 25
22	A9/46v	MFG_R_7_4	6	42, 44, 14	145	A40rv	IPL_L_6_6	2	-53, -31, 23
23	A8vl	MFG_L_7_5	7	-33, 23, 45	146	A40rv	IPL_R_6_6	2	55, -26, 26
24	A8vl	MFG_R_7_5	6	42, 27, 39	147	A7m	PCun_L_4_1	6	-5, -63, 51
25	A6vl	MFG_L_7_6	3	-32, 4, 55	148	A7m	PCun_R_4_1	6	6, -65, 51
26	A6vl	MFG_R_7_6	3	34, 8, 54	149	A5m	PCun_L_4_2	2	-8, -47, 57
27	A10l	MFG_L_7_7	5	-26, 60, -6	150	A5m	PCun_R_4_2	3	7, -47, 58
28	A10l	MFG_R_7_7	6	25, 61, -4	151	dmPOS	PCun_L_4_3	1	-12, -67, 25
29	A44d	IFG_L_6_1	6	-46, 13, 24	152	dmPOS	PCun_R_4_3	1	16, -64, 25
30	A44d	IFG_R_6_1	3	45, 16, 25	153	A31	PCun_L_4_4	7	-6, -55, 34
31	IFS	IFG_L_6_2	6	-47, 32, 14	154	A31	PCun_R_4_4	7	6, -54, 35
32	IFS	IFG_R_6_2	6	48, 35, 13	155	A1/2/3ulhf	PoG_L_4_1	2	-50, -16, 43
33	A45c	IFG_L_6_3	7	-53, 23, 11	156	A1/2/3ulhf	PoG_R_4_1	2	50, -14, 44

34	A45c	IFG_R_6_3	7	54, 24, 12	157	A1/2/3tonI	PoG_L_4_2	2	-56, -14, 16
35	A45r	IFG_L_6_4	7	-49, 36, -3	158	A1/2/3tonI	PoG_R_4_2	2	56, -10, 15
36	A45r	IFG_R_6_4	6	51, 36, -1	159	A2	PoG_L_4_3	3	-46, -30, 50
37	A44op	IFG_L_6_5	4	-39, 23, 4	160	A2	PoG_R_4_3	2	48, -24, 48
38	A44op	IFG_R_6_5	4	42, 22, 3	161	A1/2/3tru	PoG_L_4_4	2	-21, -35, 68
39	A44v	IFG_L_6_6	4	-52, 13, 6	162	A1/2/3tru	PoG_R_4_4	2	20, -33, 69
40	A44v	IFG_R_6_6	4	54, 14, 11	163	G	INS_L_6_1	2	-36, -20, 10
41	A14m	OrG_L_6_1	7	-7, 54, -7	164	G	INS_R_6_1	2	37, -18, 8
42	A14m	OrG_R_6_1	7	6, 47, -7	165	vIa	INS_L_6_2	8	-32, 14, -13
43	A12/47o	OrG_L_6_2	7	-36, 33, -	166	vIa	INS_R_6_2	6	33, 14, -13
44	A12/47o	OrG_R_6_2	7	40, 39, -14	167	dIa	INS_L_6_3	4	-34, 18, 1
45	A11l	OrG_L_6_3	5	-23, 38, -	168	dIa	INS_R_6_3	4	36, 18, 1
46	A11l	OrG_R_6_3	6	23, 36, -18	169	vId/vIg	INS_L_6_4	4	-38, -4, -9
47	A11m	OrG_L_6_4	5	-6, 52, -19	170	vId/vIg	INS_R_6_4	4	39, -2, -9
48	A11m	OrG_R_6_4	5	6, 57, -16	171	dIg	INS_L_6_5	2	-38, -8, 8
49	A13	OrG_L_6_5	5	-10, 18, -	172	dIg	INS_R_6_5	2	39, -7, 8
50	A13	OrG_R_6_5	5	9, 20, -19	173	dId	INS_L_6_6	4	-38, 5, 5
51	A12/47l	OrG_L_6_6	7	-41, 32, -9	174	dId	INS_R_6_6	4	38, 5, 5
52	A12/47l	OrG_R_6_6	7	42, 31, -9	175	A23d	CG_L_7_1	7	-4, -39, 31
53	A4hf	PrG_L_6_1	2	-49, -8, 39	176	A23d	CG_R_7_1	7	4, -37, 32
54	A4hf	PrG_R_6_1	2	55, -2, 33	177	A24rv	CG_L_7_2	8	-3, 8, 25
55	A6cdl	PrG_L_6_2	3	-32, -9, 58	178	A24rv	CG_R_7_2	8	5, 22, 12
56	A6cdl	PrG_R_6_2	3	33, -7, 57	179	A32p	CG_L_7_3	7	-6, 34, 21
57	A4ul	PrG_L_6_3	2	-26, -25,	180	A32p	CG_R_7_3	4	5, 28, 27
58	A4ul	PrG_R_6_3	2	34, -19, 59	181	A23v	CG_L_7_4	7	-8, -47, 10
59	A4t	PrG_L_6_4	2	-13, -20,	182	A23v	CG_R_7_4	1	9, -44, 11
60	A4t	PrG_R_6_4	2	15, -22, 71	183	A24cd	CG_L_7_5	4	-5, 7, 37
61	A4tl	PrG_L_6_5	4	-52, 0, 8	184	A24cd	CG_R_7_5	4	4, 6, 38
62	A4tl	PrG_R_6_5	4	54, 4, 9	185	A23c	CG_L_7_6	4	-7, -23, 41
63	A6cvl	PrG_L_6_6	3	-49, 5, 30	186	A23c	CG_R_7_6	4	6, -20, 40
64	A6cvl	PrG_R_6_6	3	51, 7, 30	187	A32sg	CG_L_7_7	7	-4, 39, -2
65	A1/2/3ll	PCL_L_2_1	4	-8, -38, 58	188	A32sg	CG_R_7_7	7	5, 41, 6
66	A1/2/3ll	PCL_R_2_1	2	10, -34, 54	189	cLinG	MVOcC_L_5_	1	-11, -82, -11
67	A4ll	PCL_L_2_2	2	-4, -23, 61	190	cLinG	MVOcC_R_5_	1	10, -85, -9
68	A4ll	PCL_R_2_2	2	5, -21, 61	191	rCunG	MVOcC_L_5_	1	-5, -81, 10
69	A38m	STG_L_6_1	5	-32, 14, -	192	rCunG	MVOcC_R_5_	1	7, -76, 11
70	A38m	STG_R_6_1	5	31, 15, -34	193	cCunG	MVOcC_L_5_	1	-6, -94, 1
71	A41/42	STG_L_6_2	2	-54, -32,	194	cCunG	MVOcC_R_5_	1	8, -90, 12
72	A41/42	STG_R_6_2	2	54, -24, 11	195	rLinG	MVOcC_L_5_	1	-17, -60, -6
73	TE1.0/T	STG_L_6_3	2	-50, -11, 1	196	rLinG	MVOcC_R_5_	1	18, -60, -7
74	TE1.0/T	STG_R_6_3	2	51, -4, -1	197	vmPOS	MVOcC_L_5_	1	-13, -68, 12
75	A22c	STG_L_6_4	2	-62, -33, 7	198	vmPOS	MVOcC_R_5_	1	15, -63, 12
76	A22c	STG_R_6_4	2	66, -20, 6	199	mOccG	LOcC_L_4_1	1	-31, -89, 11
77	A38l	STG_L_6_5	5	-45, 11, -	200	mOccG	LOcC_R_4_1	1	34, -86, 11
78	A38l	STG_R_6_5	5	47, 12, -20	201	V5/MT+	LOcC_L_4_2	3	-46, -74, 3
79	A22r	STG_L_6_6	7	-55, -3, -10	202	V5/MT+	LOcC_R_4_2	1	48, -70, -1
80	A22r	STG_R_6_6	7	56, -12, -5	203	OPC	LOcC_L_4_3	1	-18, -99, 2
81	A21c	MTG_L_4_1	7	-65, -30, -	204	OPC	LOcC_R_4_3	1	22, -97, 4
82	A21c	MTG_R_4_1	6	65, -29, -	205	iOccG	LOcC_L_4_4	1	-30, -88, -12

83	A21r	MTG_L_4_2	7	-53, 2, -30	206	iOccG	LOcC_R_4_4	1	32, -85, -12
84	A21r	MTG_R_4_2	7	51, 6, -32	207	msOccG	LOcC_L_2_1	1	-11, -88, 31
85	A37dl	MTG_L_4_3	3	-59, -58, 4	208	msOccG	LOcC_R_2_1	1	16, -85, 34
86	A37dl	MTG_R_4_3	3	60, -53, 3	209	lsOccG	LOcC_L_2_2	1	-22, -77, 36
87	aSTS	MTG_L_4_4	7	-58, -20, -9	210	lsOccG	LOcC_R_2_2	1	29, -75, 36
88	aSTS	MTG_R_4_4	7	58, -16, -	211	mAmyg	Amyg_L_2_1	8	-19, -2, -20
89	A20iv	ITG_L_7_1	5	-45, -26, -	212	mAmyg	Amyg_R_2_1	8	19, -2, -19
90	A20iv	ITG_R_7_1	5	46, -14, -	213	lAmyg	Amyg_L_2_2	8	-27, -4, -20
91	A37elv	ITG_L_7_2	3	-51, -57, -	214	lAmyg	Amyg_R_2_2	8	28, -3, -20
92	A37elv	ITG_R_7_2	3	53, -52, -	215	rHipp	Hipp_L_2_1	8	-22, -14, -19
93	A20r	ITG_L_7_3	5	-43, -2, -41	216	rHipp	Hipp_R_2_1	8	22, -12, -20
94	A20r	ITG_R_7_3	5	40, 0, -43	217	cHipp	Hipp_L_2_2	8	-28, -30, -10
95	A20il	ITG_L_7_4	7	-56, -16, -	218	cHipp	Hipp_R_2_2	8	29, -27, -10
96	A20il	ITG_R_7_4	5	55, -11, -	219	vCa	BG_L_6_1	8	-12, 14, 0
97	A37vl	ITG_L_7_5	3	-55, -60, -6	220	vCa	BG_R_6_1	8	15, 14, -2
98	A37vl	ITG_R_7_5	3	54, -57, -8	221	GP	BG_L_6_2	8	-22, -2, 4
99	A20cl	ITG_L_7_6	6	-59, -42, -	222	GP	BG_R_6_2	8	22, -2, 3
100	A20cl	ITG_R_7_6	6	61, -40, -	223	NAC	BG_L_6_3	8	-17, 3, -9
101	A20cv	ITG_L_7_7	5	-55, -31, -	224	NAC	BG_R_6_3	8	15, 8, -9
102	A20cv	ITG_R_7_7	5	54, -31, -	225	vmPu	BG_L_6_4	8	-23, 7, -4
103	A20rv	FuG_L_3_1	5	-33, -16, -	226	vmPu	BG_R_6_4	8	22, 8, -1
104	A20rv	FuG_R_3_1	5	33, -15, -	227	dCa	BG_L_6_5	8	-14, 2, 16
105	A37mv	FuG_L_3_2	1	-31, -64, -	228	dCa	BG_R_6_5	8	14, 5, 14
106	A37mv	FuG_R_3_2	1	31, -62, -	229	dlPu	BG_L_6_6	8	-28, -5, 2
107	A37lv	FuG_L_3_3	3	-42, -51, -	230	dlPu	BG_R_6_6	8	29, -3, 1
108	A37lv	FuG_R_3_3	1	43, -49, -	231	mPFtha	Tha_L_8_1	8	-7, -12, 5
109	A35/36r	PhG_L_6_1	5	-27, -7, -34	232	mPFtha	Tha_R_8_1	8	7, -11, 6
110	A35/36r	PhG_R_6_1	5	28, -8, -33	233	mPMtha	Tha_L_8_2	8	-18, -13, 3
111	A35/36c	PhG_L_6_2	5	-25, -25, -	234	mPMtha	Tha_R_8_2	8	12, -14, 1
112	A35/36c	PhG_R_6_2	1	26, -23, -	235	Stha	Tha_L_8_3	8	-18, -23, 4
113	TL	PhG_L_6_3	1	-28, -32, -	236	Stha	Tha_R_8_3	8	18, -22, 3
114	TL	PhG_R_6_3	1	30, -30, -	237	rTtha	Tha_L_8_4	8	-7, -14, 7
115	A28/34	PhG_L_6_4	5	-19, -12, -	238	rTtha	Tha_R_8_4	8	3, -13, 5
116	A28/34	PhG_R_6_4	5	19, -10, -	239	PPtha	Tha_L_8_5	8	-16, -24, 6
117	TI	PhG_L_6_5	5	-23, 2, -32	240	PPtha	Tha_R_8_5	8	15, -25, 6
118	TI	PhG_R_6_5	5	22, 1, -36	241	Otha	Tha_L_8_6	8	-15, -28, 4
119	TH	PhG_L_6_6	1	-17, -39, -	242	Otha	Tha_R_8_6	8	13, -27, 8
120	TH	PhG_R_6_6	1	19, -36, -	243	cTtha	Tha_L_8_7	8	-12, -22, 13
121	rpSTS	pSTS_L_2_1	7	-54, -40, 4	244	cTtha	Tha_R_8_7	8	10, -14, 14
122	rpSTS	pSTS_R_2_1	7	53, -37, 3	245	lPFtha	Tha_L_8_8	8	-11, -14, 2
123	cpSTS	pSTS_L_2_2	4	-52, -50,	246	lPFtha	Tha_R_8_8	8	13, -16, 7

1: Visual network; 2: Somatomotor network; 3: Dorsal attention network; 4: Ventral attention network; 5: Limbic network; 6: Frontoparietal network; 7: Default mode network; 8: Subcortical network.

References

[1]. Shen, X., *et al.* Using connectome-based predictive modeling to predict individual behavior from brain connectivity. *Nat Protoc* **12**, 506-518 (2017).

[2]. Rosenberg, M.D., *et al.* A neuromarker of sustained attention from whole-brain functional connectivity. *Nat Neurosci* **19**, 165-171 (2016).

[3]. Beaty, R.E., *et al.* Robust prediction of individual creative ability from brain functional connectivity. *Proc Natl Acad Sci U S A* **115**, 1087-1092 (2018).

[4]. Finn, E.S., *et al.* Functional connectome fingerprinting: identifying individuals using patterns of brain connectivity. *Nat Neurosci* **18**, 1664-1671 (2015).

[5]. Greene, A.S., Gao, S., Scheinost, D. & Constable, R.T. Task-induced brain state manipulation improves prediction of individual traits. *Nature communications* **9**, 2807 (2018).

[6]. Shafto, M.A., *et al.* The Cambridge Centre for Ageing and Neuroscience (Cam-CAN) study protocol: a cross-sectional, lifespan, multidisciplinary examination of healthy cognitive ageing. *BMC neurology* **14**, 204 (2014).

[7]. Taylor, J.R., *et al.* The Cambridge Centre for Ageing and Neuroscience (Cam-CAN) data repository: Structural and functional MRI, MEG, and cognitive data from a cross-sectional adult lifespan sample. *Neuroimage* **144**, 262-269 (2017).

[8]. Tsvetanov, K.A., *et al.* Extrinsic and Intrinsic Brain Network Connectivity Maintains Cognition across the Lifespan Despite Accelerated Decay of Regional Brain Activation. *J Neurosci* **36**, 3115-3126 (2016).

[9]. Geerligs, L. & Henson, R.N. Functional connectivity and structural covariance between regions of interest can be measured more accurately using multivariate distance correlation. *NeuroImage* **135**, 16-31 (2016).

[10]. Behzadi, Y., Restom, K., Liau, J. & Liu, T.T. A component based noise correction method (CompCor) for BOLD and perfusion based fMRI. *Neuroimage* **37**, 90-101 (2007).

[11]. Geerligs, L., Tsvetanov, K.A., Henson, R.N. & Gam-CAN. Challenges in measuring individual differences in functional connectivity using fMRI: The case of healthy aging. *Human Brain Mapping* **38**, 4125-4156 (2017).

[12]. Elliott, M.L., *et al.* General functional connectivity: Shared features of resting-state and task fMRI drive reliable and heritable individual differences in functional brain networks. *Neuroimage* **189**, 516-532 (2019).

[13]. Jiang, R., *et al.* Task-induced brain connectivity promotes the detection of individual differences in brain-behavior relationships. *NeuroImage* **207**, 116370 (2020).

[14]. Cui, Z., *et al.* Individual Variation in Functional Topography of Association Networks in Youth. *Neuron* **106**, 340-353 e348 (2020).

[15]. Finn, E.S., *et al.* Can brain state be manipulated to emphasize individual differences in functional connectivity? *Neuroimage* **160**, 140-151 (2017).
